# Complete Rescue of HTLV-1_p12KO_ Infectivity by Depletion of Monocytes Together with NK and CD8^+^ T Cells

**DOI:** 10.3390/pathogens13040292

**Published:** 2024-03-29

**Authors:** Anna Gutowska, Sarkis Sarkis, Mohammad Arif Rahman, Katherine C. Goldfarbmuren, Ramona Moles, Massimiliano Bissa, Melvin Doster, Robyn Washington-Parks, Katherine McKinnon, Isabela Silva de Castro, Luca Schifanella, Genoveffa Franchini, Cynthia A. Pise-Masison

**Affiliations:** 1 Animal Models and Retroviral Vaccines Section, Center for Cancer Research, National Cancer Institute, Bethesda, MD 20892, USA; anna.gutowska@nih.gov (A.G.); sarkis.sarkis@nih.gov (S.S.); isabela.silvadecastro@nih.gov (I.S.d.C.);; 2 Vaccine Branch, Center for Cancer Research, National Cancer Institute, Bethesda, MD 20892, USA; kate.goldfarbmuren@nih.gov; 3 Advanced Biomedical Computational Science, Frederick National Laboratory for Cancer Research, Leidos Biomedical Research, Inc., Frederick, MD 21702, USA; 4 Vaccine Branch Flow Cytometry Core, National Cancer Institute, Bethesda, MD 20892, USA; mckinnonkm@mail.nih.gov

**Keywords:** HTLV-1, NK cells, CD8, chemokines, cytokines, Adult-T-cell Leukemia/Lymphoma, ATLL, HTLV-1 Associated Myelopathy/Tropical Spastic Paraparesis, HAM/TSP

## Abstract

The transient depletion of monocytes alone prior to exposure of macaques to HTLV-1 enhances both HTLV-1_WT_ (wild type) and HTLV-1_p12KO_ (Orf-1 knockout) infectivity, but seroconversion to either virus is not sustained over time, suggesting a progressive decrease in virus expression. These results raise the hypotheses that either HTLV-1 persistence depends on a monocyte reservoir or monocyte depletion provides a transient immune evasion benefit. To test these hypotheses, we simultaneously depleted NK cells, CD8^+^ T cells, and monocytes (triple depletion) prior to exposure to HTLV-1_WT_ or HTLV-1_p12KO_. Remarkably, triple depletion resulted in exacerbation of infection by both viruses and complete rescue of HTLV-1_p12KO_ infectivity. Following triple depletion, we observed rapid and sustained seroconversion, high titers of antibodies against HTLV-1 p24Gag, and frequent detection of viral DNA in the blood and tissues of all animals when compared with depletion of only CD8^+^ and NK cells, or monocytes alone. The infection of macaques with HTLV-1_WT_ or HTLV-1_p12KO_ was associated with higher plasma levels of IL-10 after 21 weeks, while IL-6, IFN-γ, IL-18, and IL-1β were only elevated in animals infected with HTLV-1_WT_. The repeat depletion of monocytes, NK, and CD8^+^ cells seven months following the first exposure to HTLV-1 did not further exacerbate viral replication. These results underscore the contribution of monocytes in orchestrating anti-viral immunity. Indeed, the absence of *orf-1* expression was fully compensated by the simultaneous depletion of CD8^+^ T cells, NK cells, and monocytes, underlining the primary role of *orf-1* in hijacking host immunity.

## 1. Introduction

Human T-cell Leukemia Virus type-1 (HTLV-1) is a Deltaretrovirus first identified in 1980 [1]. At least 5–10 million individuals are estimated to be infected with HTLV-1 worldwide based on curated data from regions where the virus is endemic, though a more accurate range is believed to be higher, as many affected countries do not screen for HTLV in the blood donor information that is used to generate these data [2]. While the majority of infected individuals remain asymptomatic, 2–5% of HTLV-1-positive patients develop the fatal CD4^+^ T-cell malignancy Adult-T-cell Leukemia/Lymphoma (ATLL) [1,3,4,5,6]. Although HTLV-1 infection does not represent a high global burden, HTLV-1 typically has high prevalence in the affected populations and is one of the most oncogenic human viruses [7]. HTLV-1 infection is also the causative agent of the progressive neurodegenerative disorder HTLV-1-Associated Myelopathy/Tropical Spastic Paraparesis (HAM/TSP) [8,9] and is associated with a number of inflammatory diseases, such as infectious dermatitis, HTLV-1-associated arthropathy, uveitis, and polymyositis [10,11,12]. Immune dysregulation is a common feature in HTLV-1 infection [13,14,15,16,17,18,19,20], and although there is a strong innate and adaptive response to the virus, it results in a lifelong persistent infection of primarily T lymphocytes [21,22,23,24,25,26]. This is likely linked to the ability of the virus to counteract host responses [27,28].

A key viral gene shown to prevent host immune recognition is the open reading frame-1 (*orf-1*) gene, which encodes the non-structural protein product p12 and its cleavage product, p8 [29,30,31]. Several in vitro studies demonstrated that the p12 and p8 proteins counteract Natural Killer (NK) cell [32,33] and CD8^+^ cytotoxic T-lymphocyte (CTL) responses [24], increase T-cell proliferation [34,35], and enhance viral transmission [36,37,38]. The p12 protein decreases the cell surface expression of major histocompatibility complex class I (MHC-I) by binding to newly synthesized MHC-I heavy chain in the endoplasmic reticulum before it interacts with β2-microglobulin, causing it to instead be rerouted to the proteasome for degradation [39]. Reduced cell surface MHC-I expression, in turn, reduces CTL recognition of infected cells [24,32]. Since reduced MHC-I expression on infected cells could make them susceptible to NK cell lysis [40], the p12 protein also counters NK cell recognition by decreasing the expression of ICAM-1 and ICAM-2 [32,33]. The p12 protein also contributes to infection through the stimulation of T-cell proliferation by activating nuclear factor of T cells (NFAT) proteins [41,42,43], stimulating cytosolic calcium through the binding of calreticulin and calnexin [44] and binding to the interleukin 2 receptor (IL2R) β and γ chains in the ER to promote STAT5 activation, reducing the requirement threshold for IL-2 for T-cell growth [35]. In addition, the p8 protein enhances HTLV-1 transmission and signaling by inducing cellular conduits and increasing T-cell adhesiveness [36,45]. These cellular conduits enable the transfer of the HTLV-1 Gag, Env, Tax, and p8 proteins to the target cells [36]. Using a quantitative flow cytometry-based method, p8 was calculated to be transferred in an actin polymerization-dependent process to about 5% of recipient cells after 5 min of co-culture [46]. In addition, when HTLV-1-transformed cells were treated with cytarabine to reduce cellular conduits, virus production and transmission were decreased [38].

While Orf-1 proteins are dispensable for viral replication in vitro [47,48,49,50], they are essential for viral persistence in vivo in the rhesus macaque model [24,47]. The infectious molecular clone engineered to specifically knock out the expression of only the *orf-1*-encoded proteins, p12 and p8 (HTLV-1_p12KO_), could not sustain infection in macaques, whereas wild type HTLV-1 (HTLV-1_WT_) caused persistent infection in 75% of the animals [24,47]. The importance of the *orf-1* gene is also supported by its evolutionary maintenance in the vast majority of HTLV-1 subtype A genomic clones sequenced to date [24,51]. In a study analyzing *orf-1* sequences from 231 patients chronically infected with HTLV-1 (144 HAM/TSP, 41 ATLL, and 46 carriers) from the Kagoshima region, Japan, 8 HAM/TSP and 2 ATLL patients had viral clones that encoded truncated p12 protein (amino acid 82 or 87) [51]. Interestingly, these truncated proteins retained the functional domains for calcineurin binding, IL-2 receptor beta- and gamma-chain binding, leucine zippers, and amino terminal cleavage sites. In another study in chronically infected individuals, none of the 1600 *orf-1* clones sequenced from 160 HTLV-1-infected patients (HAM/TSP and carriers) from various geographical regions had premature stop codons [24].

In a recent study to investigate the role of NK cells, CTLs, and monocytes/macrophages in controlling HTLV-1 infection, we depleted specific cell populations in the rhesus macaque model prior to infection with either HTLV-1_WT_ (WT) or HTLV-1_p12KO_ (p12KO) [52]. Interestingly, the depletion of either CD8^+^ cells alone or NK and CD8^+^ cells together enhanced infection with HTLV-1_WT_, causing the seroconversion of all six animals by 4 weeks after baseline, compared with week 21 for replete control animals. In contrast, the depletion of CD8^+^ cells resulted in the seroconversion of only one of four animals, but the depletion of both NK and CD8^+^ cells resulted in the seroconversion of all four animals by week 8, even in animals exposed to HTLV-1_p12KO_ virus. All animals that seroconverted (exposed to either WT or p12KO) had detectable viral DNA according to nested PCR in both the periphery and tissues. Importantly, we did not detect viral reversion in animals infected with HTLV-1_p12KO_. Together, the results indicate that depleting NK and CD8^+^ cells early in infection enhances the establishment of HTLV-1 infection. In addition, similar to what was seen in a humanized mouse model [53], once HTLV-1 infection is established, the expression of *orf-1* is not required to maintain infection, since this is primarily conducted through the proliferation and division of cells carrying proviral DNA.

Understanding the host innate and adaptive responses to HTLV-1 is a critical step toward designing a preventive HTLV-1 vaccine. While the early evasion of CD8^+^ and NK cell responses is important for controlling HTLV-1 infection, the role of monocytes/macrophages appears to be more complex and is still not fully understood. In a recent study, when monocytes/macrophages were depleted using Clodrosome prior to viral exposure, HTLV-1_WT_ infection was enhanced, with seroconversion in all five animals being detectable by week 8 [52]. However, by week 21 post-viral exposure, reactivity to viral antigens dramatically decreased in four of the five animals. In contrast, monocyte depletion prior to exposure to HTLV-1_p12KO_, a virus unable to infect monocytes and dendritic cells in vitro and immunocompetent macaques in vivo, did not fully rescue infectivity. Seroconversion was observed in only one of five animals and also declined at week 21 after exposure to HTLV-1_p12KO_. Collectively, these results suggest that either the early engagement of monocytes is necessary to establish long-term, productive HTLV-1 infection or monocytes play a vital role in orchestrating effective anti-viral immunity. Here, we demonstrate that depleting NK cells, CD8^+^ cells, and monocytes prior to viral exposure results in robust and sustained infection with HTLV-1_WT_. In addition, the simultaneous depletion of all three cell subsets resulted in a complete rescue of HTLV-1_p12KO_ infectivity, favoring the hypothesis that a primary role of *orf-1* in infection is to hijack early innate as well as adaptive host immunity.

## 2. Materials and Methods

### 2.1. Ethics Statement

The animals used in this study were colony-bred Indian rhesus macaques (Macaca mulatta) obtained from Covance Research Products Inc. (Alice, TX). Animals were housed at the National Institutes of Health, Bethesda, MD. Animals were cared for in accordance with American Association for the Accreditation of Laboratory Animal Care (AAALAC) standards in an AAALAC-accredited facility (OLAW; Animal Welfare Assurance A4149-01). All animal procedures reported in this study that were performed by NCI-CCR affiliated staff were approved by the NCI Animal Care and Use Committee (ACUC) and were in accordance with federal regulatory requirements and standards. All components of the intramural NIH ACU program are accredited by AAALAC International. Steps were taken to ensure the welfare of the animals and minimize discomfort of all animals used in this study. Animals were fed daily with a fresh diet of primate biscuits, fruit, peanuts, and other food items to maintain body weight or normal growth. Animals were monitored for mental health and provided with physical enrichment, including sanitized toys, destructible enrichment (cardboard and other paper products), and audio and visual stimulation. 

### 2.2. Animal Inoculation and Treatments

Ten male rhesus macaques from a prior study that were confirmed to be uninfected by SIV/SHIV (Table S1), as demonstrated by several consecutive negative PCR tests, were enrolled in this study. Animals were treated for three days with depleting anti-CD8 monoclonal antibody clone M-T807R1 (targeting the α/α chain). M-T807R1 was purchased from the NHP Reagent Resource Program (University of Massachusetts Medical School, Worcester, MA, USA) and injected intravenously at 5 mg/kg per day for three consecutive days prior to virus inoculation. One day prior to virus inoculation, all animals were also treated intravenously once with 5 mg/kg of Clodrosome (Encapsula NanoSciences LLC, Brentwood, TN, USA). Animals were then inoculated intravenously with lethally γ-irradiated 729.6 B cells producing HTLV-1_WT_ or HTLV-1_p12KO_ virus. The inoculated cell number was normalized based on equivalent levels of p19Gag antigen production and on viral DNA load [24,47,52]. Animals were monitored for more than 30 weeks and then euthanized to study viral dissemination in tissues.

### 2.3. HTLV Serology, Tax and HBZ Expression, and Viral DNA Detection

Reactivity to specific viral antigens in the plasma of infected animals was detected with the use of a commercial HTLV-1 Western immunoblot assay (GeneLabs Diagnostics, Redwood City, CA, USA). HTLV-1 p24 antibodies in plasma samples from macaques were detected and quantified against purified HTLV-1 p24 protein by using an ELISA assay (Advanced BioScience Laboratories, Inc., Rockville, MD, USA) according to the manufacturer’s instructions. The plate was read at 450 nm (E-max reader; Molecular Devices).

Total cellular RNA was extracted from PBMCs (weeks 7, 21, and 33 and necropsy) and spleen from all triple-depleted animals by using an RNeasy plus mini kit (cat. # 74034; Qiagen, Germantown, MD, USA) by following the manufacturer’s instructions. RNA samples were quantified using a Nanodrop ND-2000 apparatus (Thermo Fisher Scientific, Grand Island, NY, USA) and were subjected to reverse transcription by using a QuantiTect Reverse transcription Kit according to the manufacturer’s instructions (Qiagen, Germantown, MD, USA; cat. # 205311). Nested PCR was performed on cDNA samples for the detection of the *tax* and *hbz* genes by using the primers sHBZ (sHBZ F: 5′CCTCAGGGCTGTTTCGATG-3′; sHBZ R: 5′-TAGCCATCAATCCCCAACTC-3′; HBZ Nested: 5′-TTCGATGCTTGCCTGTGCCA3′) and Tax/Rex (Tax/Rex F: 5′-ACCAACACCATGGCCCAC-3′; Tax/Rex R: 5′-AGGGTTGATTGGAACGGAAG-3′; Tax/Rex Nested: 5′-CTCGACTCCCCTCCTTCCCCA-3′). The PCR conditions used were 94 °C for 2 min, followed by 35 cycles of 94 °C for 30 s, 68 °C for 60 s, 68 °C for 60 s, a final extension at 68 °C for 7 min, and a hold at 4 °C. Platinum High Fidelity PCR SuperMix (Invitrogen, Carlsbad, AC, USA) was used according to the manufacturer’s instructions. Correctly sized amplicons were identified by 1% agarose gel electrophoresis.

Genomic DNA from PBMCs, bone marrow, bronchoalveolar lavage (BAL), and necropsy biopsies (Ileum, Colon, Thymus, Skin, Jejunum, Cortex, Pons, Lung, Spleen, Inguinal, Mesenteric, Axillary, and Lung lymph nodes) were isolated by using the DNeasy Blood and Tissue Kit (Qiagen). One hundred nanograms of DNA was used as template for the PCR amplification of the *gag* and *orf-1* genes by using the primers gag-F1 (50-GGCCAAATCCTTTCCCGTAG-30) and gag-R1(50-GTTGTGGATTGTTGGCTTGG-30), or p12-F1 (50-CCTCGCCCTTCCAACTGTCT-3′) and p30-R1 (50-AGGAAGGAGGGTGGAATGTT-3′). Three microliters of the PCR reaction was used as a template for nested PCR by using the following primers: gag-F2 (50-GTCCCTCCAGTTACGATTTCC-30) and gag-R2 (50-AGGGAGGAGCAAAGGTACTG-30), or p12-F2 (5′ CGCCTTCCAACTGTCTAGTATAGC-3′) and p30-R2 (5′-GGGAGTCGAGGGATAAGGAA-3′). The PCR conditions used were 94 °C for 7 min, followed by 35 cycles of 94 °C for 30 s, 55 °C for 30 s, 68 °C for 60 s, a final extension at 68 °C for 7 min, and a hold at 4 °C. Platinum High Fidelity PCR SuperMix (Invitrogen) was used according to the manufacturer’s instructions. Correctly sized amplicons were identified by 1–1.5% agarose gel electrophoresis. Sanger sequencing was carried out on the amplicons at the Center for Cancer Research Genomics Core at the National Cancer Institute, NIH, to verify HTLV-1 sequence amplification.

The HTLV-1 proviral load (PVL) was measured by real-time PCR by using 5 ng/µL of DNA and the TaqMan Universal PCR Master Mix (Applied Biosystems, Foster City, CA, USA) in the Rotor-Gene Q system (Qiagen). Standard curves were generated by amplification of RNase P gene fragment from HTLV-1-negative genomic DNA isolated from PBMCs with the use of Taqman RNase P Detection Reagents VIC (Applied Biosystems) and the HTLV-1 pX region fragment from a pABD26 molecular clone plasmid [24]. The sequences of primers and probes for the pX gene were as follows: 5′-CGGATACCCAGTCTACGTGTT-3′, 5′-CAGTAGGGCGTGACGATGTA-3′, and 3′-FAM/CTGTGTACAAGGCGACTGGTGCC-3′ [54]. The reaction conditions were 1 cycle of 2 min at 50 °C, followed by 1 cycle of 10 min at 95 °C, then 40 cycles of 15 s at 95 °C, followed by 60 s at 60 °C. HTLV-1 provirus DNA levels (PVL) were calculated by the following formula: (copies of HTLV-1 (pX)/(copies of RNase P/2) × 100 cells. Samples with provirus unquantifiable by qPCR (pvl < 0.02% PBMCs) were amplified by nested PCR to confirm the presence of viral DNA.

### 2.4. Multiplex Assay of Plasma

Cryopreserved supernatants from plasma collected from rhesus macaques at baseline, week 2, week 5, week 7, week 16, and week 21 of the study were analyzed by using the MILLIPLEX MAP Non-Human Primate Cytokine Magnetic Bead Panel kit (Millipore Sigma, St Louis, MO, USA). The following targets were assayed by following the manufacturer’s instructions: IFN-γ, IL-10, sCD40L, IL-13, IL-1β, IL-6, IL-8, MIP-1α, MIP-1β, TNFα, IL-12/23, and IL-18. After thawing the plasma on ice, 25 µL of each was loaded into the well and mixed with 25 µL of assay buffer and 25 µL of magnetic beads. The plates were incubated under agitation at 4 °C for 18 h. After washing, 25 µL of detection antibody was added to each well, and the plate was incubated; then, after the addition of 25 µL of Streptavidin-Phycoerythrin, the plate was washed and mixed with 150 µL of Sheath Fluid. The Median Fluorescent Intensity (MFI) was measured with the use of the Bio-Plex^®^ 200 system with HTF (Bio-Rad, Hercules, CA, USA).

### 2.5. Flow Cytometry Analysis of Rhesus Macaque Whole-Blood Samples

A 22-color panel was designed to examine the changes in cell phenotypes in whole blood collected from animals during the course of the study (surface staining for CD3, CD4, CD8 (two clones), CD11b, CD11c, CD14, CD16, CD20, CD25, CD28, CD45, CD49d, CD56, CD66abce, CD95, CD123, CD127, CD159 (NKG2a), CD194 (CCR4), and HLA-DR). All antibodies were selected based on cross-reactivity with rhesus macaques and fluorochrome availability. Antibody information, including clones and fluorochromes, is listed in Table S2.

Briefly, 100 μL of fresh EDTA whole blood was stained with Live/Dead Fixable Blue dye (ThermoFisher Scientific) and surface-stained at room temperature for 30 min. BD FACS Lysing solution (BD Biosciences, San Jose, CA, USA) was then used to lyse red blood cells according to the manufacturer’s instructions. Samples were washed twice with D-PBS and resuspended in 1% ultrapure formaldehyde (Tousimis, Rockville, MD, USA). Samples were acquired on a BD FACSymphony A5 analyzer using FACSDiva 8 software. Data were analyzed by using the FlowJo (version 10.6.) gating strategy as outlined in Figure S1A,B, established by using a combination of isotype and fluorescence-minus-one (FMO) controls.

### 2.6. Statistical Analysis

Correlation analyses were calculated by using the non-parametric Spearman rank correlation method. Changes with time for each variable were computed by fitting generalized estimating equations, with animal ID as a random effect. For visualization, the fold change (FC) for each animal was calculated as 100 * (week XX + 0.0001)/(baseline + 0.0001). In all performed analyses, we considered results with *p* < 0.05 to be statistically significant.

## 3. Results

### 3.1. Recovery of Cell Subsets Following Treatment of Macaques with Drugs Depleting NK Cells, CD8^+^ T Cells, and Monocytes Prior to HTLV-1 Infection

We investigated the contribution of the simultaneous depletion of monocytes/macrophages, CD8^+^ T cells, and NK cells to infection by HTLV-1_WT_ and HTLV-1_p12KO_ with the goals of better understanding the role of Orf-1 in hijacking host immunity. Three days before exposure to virus-producing cells (starting on day −3), ten macaques were administered intravenously with MT807-R1, an anti-CD8 monoclonal antibody that targets the α/α chain of NK cells and CD8^+^ lymphocytes. In addition to MT807-R1, on day −1, macaques were also given one dose of Clodrosome. On day 0, lethally irradiated HTLV-1_WT_- or HTLV-1_p12KO_-producing cells were intravenously administered, and samples were taken periodically to assess seroconversion, cell phenotype, and viral DNA level (Figure S2A).

**Figure 1:**
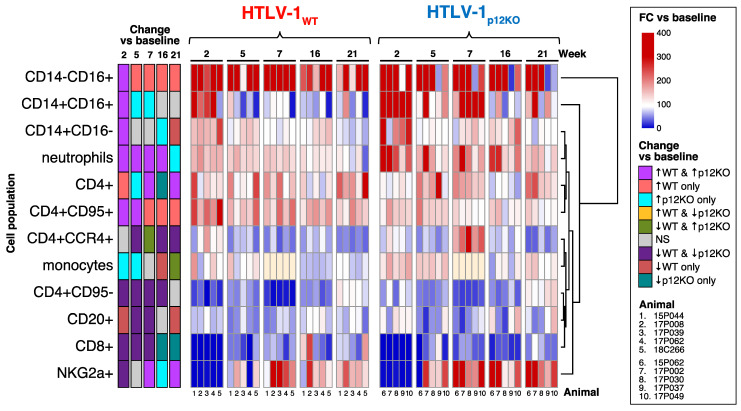
Immunophenotypic profiles of peripheral whole blood of animals infected with HTLV-1_WT_ or HTLV-1_p12KO_ after triple depletion of NK cells, CD8^+^ cells, and monocytes. The frequency of absolute CD8^+^ and CD4^+^ T-cell, B-cell (CD20^+^), and monocyte cell counts; the frequency of the three monocyte subsets of classical (CD14^+^CD16^−^), intermediate (CD14^+^CD16^+^), and non-classical (CD14^−^CD16^+^); and the frequency of NKG2a^+^ cells and neutrophils from whole blood were measured before treatment (baseline) and throughout the study (weeks 2, 5, 7, 16, and 21) in HTLV-1_WT_- or HTLV-1_p12KO_-infected macaques. Changes for each variable with time were computed with generalized estimating equations with animal ID as a random effect. For visualization, the FC for each animal was calculated as 100 * (week XX + 0.0001)/(baseline + 0.0001) and graphed as a heatmap, in which increases are indicated in red and decreases in blue. The scale is given to the right of the plot. Statistical information is shown in the first 5 columns on the left of the heatmap and is color-coded, indicating which cell populations changed with time vs. baseline (*p* < 0.05). The color scale (↑ for increase and ↓ for decrease) is given to the right of the heatmap, where gray indicates a non-significant change (*p* > 0.05) in both virus groups. No data were available for monocytes at week 7 in either group (peach). The group name and timepoints are indicated above the heatmap. Animal ID numbers are shown below the graph.

As expected, complete depletion of CD8^+^ cells and reduction in NKG2a^+^ cells to <1% after administration of M-T807R1/Clodrosome were measured at the time of virus exposure (day 0) in all animals of both groups (Figure S2B,C; Table S3). By week 7, CD8^+^ cells were detected in all animals, although still at levels below baseline levels. CD8^+^ cells recovered to frequencies indistinguishable from baseline levels by week 16 in the WT group but remained significantly decreased relative to baseline levels in the p12KO group until week 21 (Figure 1). NKG2a^+^ cells recovered by week 5 in both groups and were significantly higher relative to baseline levels from week 7 onward at all subsequent timepoints in the p12KO group and most timepoints in the WT group (Figure 1). Peripheral blood monocytes were decreased in all 10 animals, but only marginally, and recovered by week 1 (Figure S2B,C; Table S3). The p12KO group saw increased monocyte levels relative to baseline at weeks 2, 5, and 21, while the WT group had decreased monocyte levels relative to baseline at weeks 16 and 21 (Figure 1).

Because of the variation in baseline values for cell populations among the animals, generalized estimating equations were fitted to evaluate changes in cell populations for animals infected with WT compared to those with p12KO virus over the course of the study (Figure 1). At week 2 post-viral exposure, both WT and p12KO groups demonstrated a clear increase in all three monocyte populations relative to their baseline levels. Subsequently, CD14^−^CD16^+^ (non-classical) monocytes persisted at significantly higher levels relative to week 0 throughout the 21 weeks of the study only in WT-infected macaques. In contrast, CD14^+^CD16^+^ (intermediate) monocytes remained elevated at weeks 5 and 7 relative to baseline levels only in p12KO-infected macaques. CD14^+^CD16^−^ (classical) monocytes returned to baseline levels at weeks 5 and 7 in both groups, with a significant increase at week 16 in the p12KO group and a decrease at week 21 in the WT group (Figure 1). This pattern of changes in monocytes confirmed our previous findings in WT virus that chronic HTLV-1 infection is associated with a lower frequency of classical monocytes and increased frequencies of non-classical monocytes (78).

The depletion of CD8^+^ cells, NK cells, and monocytes affected the frequency of other cell populations. Neutrophils increased in both groups through week 16, while CD4^+^ T cells exhibited increases in frequency at the majority of timepoints across the study in both WT- and p12KO-infected animals. (Figure 1). CD4^+^CD95^+^ cells were elevated at weeks 2 and 5 relative to baseline levels in both groups but remained significantly higher until week 21 in the WT group only. In contrast, a marked drop in naïve CD4^+^CD95^−^ cells from weeks 2 to 16 compared with baseline levels was observed in both WT- and p12KO-infected animals. We detected a significant decrease in B-cell (CD20^+^) frequencies below baseline levels in animals infected with WT and p12KO, although this decrease was slower to start (week 5 vs. week 2) and returned to baseline levels by week 16 in the p12KO group (Figure 1). The percentage of CD4^+^CCR4^+^ T cells also decreased at weeks 5, 16, and 21 compared with baseline levels in both groups (Figure 1). However, at week 7, WT-infected animals continued to exhibit decreased frequencies of CD4^+^CCR4^+^ T cells, while the percentage of CD4^+^CCR4^+^ T cells nearly doubled in all p12KO-infected animals (Figure 1 and Figure S2B,C). Notably, increases in the percentage of CD4^+^CCR4^+^ T cells are commonly found in inflammatory diseases [55,56,57], and the finding here is consistent with our previous results indicating that p12KO virus infection is associated with a higher inflammatory profile [52].

### 3.2. Equivalent Exacerbation of HTLV-1_WT_ and HTLV-1_p12KO_ Infection by Prior Depletion of CD8^+^ T Cells, NK Cells, and Monocytes

**Figure 2:**
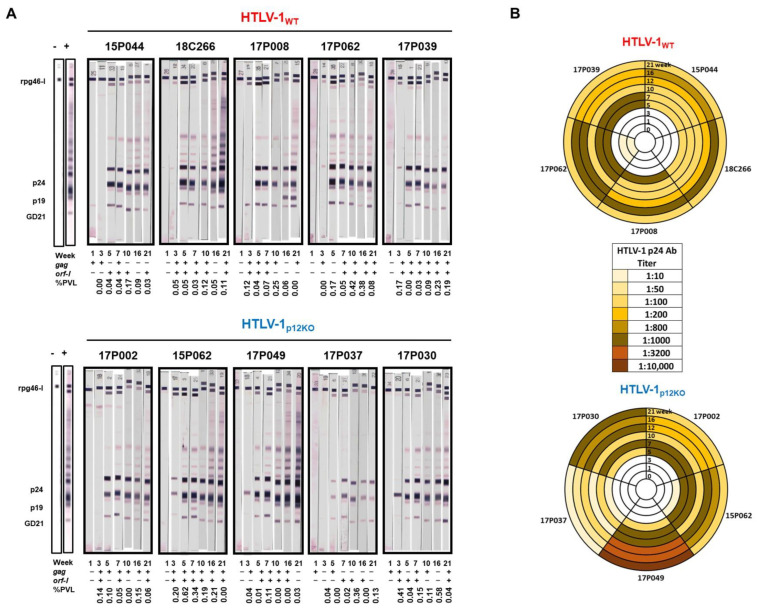
Antibody responses. (**A**) Sera from the inoculated macaques belonging to HTLV-1_WT_ and HTLV-1_p12KO_ groups were assayed at weeks 1, 3, 5, 7, 10, 16, and 21 for reactivity to HTLV-1 antigens using the kit from HTLV Blot 2.4 Western Blot Assay (MP Diagnostics, Singapore). The animal ID, inoculated viruses, and treatment are indicated above each sample. The week of serum collection is indicated below each Western blot strip. Nested PCR amplifying the *gag* and *orf-1* genes and qPCR for the assessment of HTLV-1 proviral load were performed in the DNA isolated from the PBMCs of each animal throughout the course of the study (see Material and Methods) and are indicated below each Western blot strip. The positive amplification of either *gag* or *orf-1* is symbolized by (+); the absence of amplification is symbolized by (-); the PVL is shown as the number of HTLV-1 proviral DNA copies per 100 PBMCs. (**B**) The serum HTLV-1 p24Gag antibody titer was measured for macaques belonging to the HTLV-1_WT_ (top panel) and HTLV-1_p12KO_ (bottom panel) groups at weeks 1, 3, 5, 7, 10, 12, 16, and 21. Dilutions of 1:10, 1:50, 1:100, 1:200, 1:800, 1:1000, 1:3200, and 1:10,000 were used and color-coded as reported in the figure.

As shown previously, the depletion of both NK and CD8^+^ cells or CD8^+^ cells alone prior to exposure to HTLV-1_WT_ increased susceptibility to infection (all macaques became infected compared with 75% untreated macaques) and shortened the timeline to seroconversion, with all treated animals seroconverting by week 4 [24,52]. In the case of HTLV-1_p12KO_, however, the depletion of CD8^+^ and NK cells resulted in later seroconversion in all animals at week 8, and the depletion of CD8^+^ cells alone resulted in the seroconversion of only one of four animals. These results underscore the importance of Orf-1 function in NK activity. Clodrosome treatment alone to deplete monocytes resulted in the seroconversion of all five macaques exposed to HTLV-1_WT,_ but only one out the of five exposed to HTLV-1_p12KO_. However, antibody titers against HTLV-1 p24Gag were not sustained in either group [52]. In the current triple-depletion study, by week 5, we observed full and sustained seroconversion in five out of five macaques exposed to HTLV-1_WT_ and those exposed to HTLV-1_p12KO_ (Figure 2A,B). The p24Gag antibody titers increased over the course of the study until week 16, mirroring the results obtained with the Western blot strips (Figure 2A). Interestingly, by week 21, p24Gag antibody titers decreased in all WT-infected macaques to a titer of 1:100 (Figure 2B). In contrast, p24Gag antibody titers displayed a varied pattern in the p12KO group, decreasing in three of the five infected macaques and increasing in two of the five animals, reaching a titer of 1:10,000 in animal 17P049 (Figure 2B).

**Figure 3:**
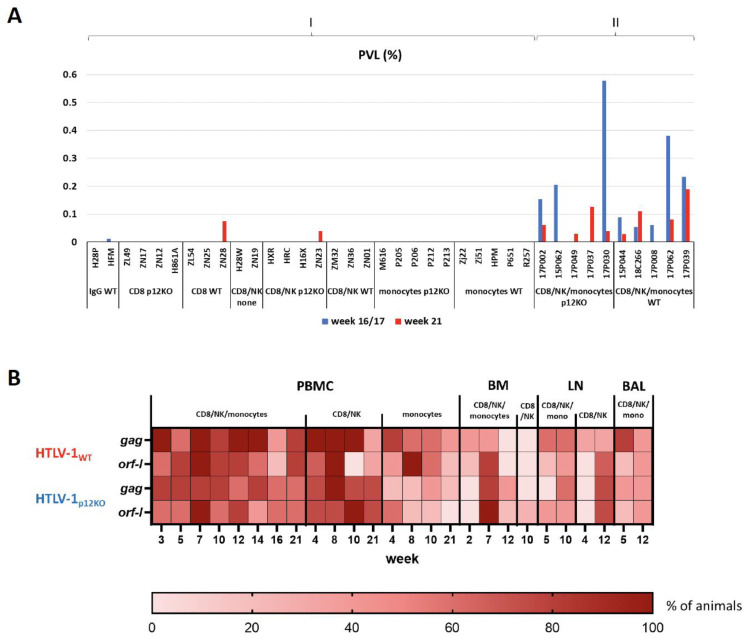
Detection of viral DNA. (**A**) Genomic DNA was isolated from PBMCs from macaques infected with HTLV-1_WT_ or HTLV-1_p12KO_ virus at weeks 16/17 and 21. Comparison of HTLV-1 proviral load (PVL) between animals from our previous study (group I), infected with HTLV-1_WT_ or HTLV-1_p12KO_ and not depleted, (IgG), CD8^+^ cell-depleted (CD8β255R1), CD8+/NK cell-depleted (M-T807R1), or monocyte only-depleted (Clodrosome) [52] and triple-depleted animals in this study (group II; infected with HTLV-1_WT_ and HTLV-1_p12KO_). The animal ID and group names are indicated below the graph. PVL is given as a percentage (the number of HTLV-1 proviral DNA copies per 100 PBMCs) at weeks 16/17 (blue) and 21 (red) of the study. (**B**) Distribution of the HLTV-1 *gag* and *orf-1* genes. Genomic DNA was isolated from peripheral blood mononuclear cells (PBMCs), bone marrow (BM), lymph nodes (LNs) and bronchoalveolar lavage (BAL) after treatment with M-T807R1/Clodrosome (CD8/NK/monocytes), with M-T807R1 (CD8/NK) alone or with Clodrosome (monocytes) alone. Nested PCR for *gag* and *orf-1* was performed (see Materials and Methods). The heatmap represents the distribution of genes. The timepoints of the study (weeks) and the scale indicating the percentage of PCR-positive animals are shown under the graph.

Although triple depletion did not significantly impact the timing of the antibody response to viral infection compared with M-T807R1 alone, a clear difference in the ability to detect and quantify viral DNA was found. Nine of ten macaques were positive according to nested PCR for the *gag* and/or *orf-1* genes at week 3 post-infection, except animal 17P037 from the p12KO group (Figure 2A). This animal also had the lowest p24Gag titer (Figure 2B). We did see variations in *gag* vs. *orf-1* positivity, perhaps due to differences in primer sensitivity, length of the amplicon, and/or presence of defective proviruses. The depletion of monocytes alone or CD8^+^ and NK cells (double depletion) resulted in unmeasurable levels of viral DNA in genomic DNA isolated from peripheral blood mononuclear cell (PBMC) samples from seroconverted animals [52], whereas in the current study, using the identical viral DNA measurement method, viral levels were detected in all triple-depleted animals (Figure 2A). To directly compare proviral loads (PVLs), quantitative real-time PCR was performed on genomic DNA isolated from PBMCs at two timepoints (weeks 16 and 21) from animals of our previous study [52] and triple-depleted animals (Figure 3A). Single-agent treatment resulted in predominantly undetectable viral DNA levels (Figure 3A, group I). In contrast, DNA viral levels were measurable in all triple-depleted macaques for at least one of the timepoints (Figure 3A, group II). No significant correlations between changes in cell counts or cell frequencies and HTLV-1 viral DNA in blood were found.

Next, we performed nested PCR to detect viral DNA in different tissue compartments of triple- vs. double-depleted animals. A heatmap plot indicates the proportion of animals positive for *gag* and *orf-1* DNA detected in PBMCs, bone marrow (BM), lymph nodes (LNs), and bronchoalveolar lavage (BAL) collected during the course of the study (Figure 3B). The frequency of detection of the HTLV-1 *gag* and *orf-1* DNA in PBMCs, BM, and LNs was similar in triple- and double-depleted animals (Figure 3B). Of note, the sequencing of PCR products confirmed no mutation or reversion of the input virus.

### 3.3. Immune Mediators in the Plasma of HTLV-1-Infected Macaques

**Figure 4:**
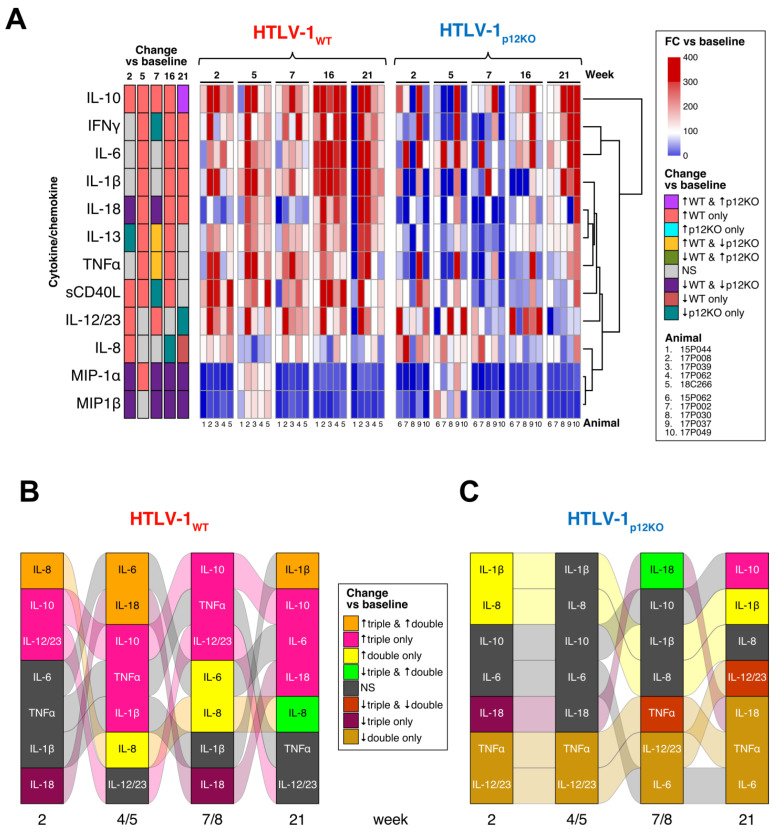
Cytokine production in plasma of HTLV-1-infected macaques. Cytokine levels were analyzed by using the MILLIPLEX MAP Non-Human Primate Cytokine Magnetic Bead Panel kit (Millipore Sigma). (**A**) Changes relative to baseline for each variable were computed with generalized estimating equations with animal ID as a random effect. For visualization, FC vs. baseline for each animal was calculated as 100 * (week XX + 0.0001)/(baseline + 0.0001) and graphed as a heatmap, where increases are shown in red and decreases in blue. The scale is given to the right of the plot. Statistical information for changes vs. baseline is shown in the 5 columns on the left of the heatmap and is color-coded, indicating which cytokines changed over time vs. baseline (*p* < 0.05), where gray indicates a non-significant change (*p* > 0.05) in both groups. The group name and timepoints are indicated above the heatmap, the cytokine/chemokine names on the left of the plot, and animal ID numbers below the graph. Cytokine/chemokine changes in HTLV-1_WT_ (**B**) and HTLV-1_p12KO_ (**C**) infection. Comparison of plasma cytokine profiles in HTLV-1-infected macaques after double vs. triple depletion. Alluvial diagrams summarize the overlap between significant changes vs. baseline in cytokines for each group throughout the study. (**B**) Changes in the levels of IL-8, IL-1β, IL-10, IL-6, TNFα, IL-18, and IL-12/23 in the plasma of HTLV-1_WT_-infected animals after M-T807R1/Clodrosome (triple) or M-T807R1 (double) treatment measured at weeks indicated below the columns. **(C)** Changes in the levels of IL-8, IL-1β, IL-10, IL-6, TNFα, IL-18, and IL-12/23 in the plasma of HTLV-1_p12KO_-infected animals after M-T807R1/Clodrosome (triple) or M-T807R1 (double) treatment measured at weeks indicated below the columns. Paired analysis fitting generalized estimating equations (geeGLM) was used to determine cytokines that significantly changed over time in each group. Statistical information is represented as ↑ for increase, ↓ for decrease, and NS for not significant for *p* ≥ 0.05 in both groups. Each column is sorted according to the pattern of significant changes vs. baseline across the groups indicated. Colors represent these patterns (legend between the plots). Streams (alluvia) between columns highlight the significance/direction pattern over time for each cytokine and are colored according to the pattern at week 2 vs. baseline (leftmost column). The virus is indicated above the heatmap, and timepoints and groups are shown below the graph. Triple-depleted animals were assayed at weeks 5 and 7, while double-depleted animals were assayed at weeks 4 and 8.

To further characterize the immune response to HTLV-1 infection, cytokine levels in plasma from all animals were measured using a multiplex non-human primate assay (Luminex). Paired analysis fitting generalized estimating equations determined which cytokines significantly changed with time relative to their baseline levels for each group (Figure 4A). MIP-1α and MIP-1β decreased after triple depletion to lower than baseline levels from week 7 to the end of the study in both the WT and p12KO groups (Figure 4A). In contrast, most cytokines measured increased in the WT group at week 5 and week 16, with IL-10, IFN-γ, IL-6, IL-1β, and IL-18 remaining elevated relative to baseline levels until week 21 (Figure 4A, Table S4). In the HTLV-1_p12KO_ group, most cytokines remained at baseline levels or decreased. IL-10 was the only cytokine to increase over baseline levels in the HTLV-1p12KO group (at week 21) (Figure 4A). We analyzed the correlation between the changes in cytokine levels and cell subsets and found distinct associations across the two virus groups (Figure S3). WT-infected animals displayed more significant associations (positive in four and negative in seven) than p12KO-infected animals (positive in one and negative in four), suggesting that a broad set of immune cells and cytokines are involved in the response to the WT virus. Indeed, in WT-infected animals, positive associations were observed between cytokines and neutrophils (IFN-γ at week 2), CD14^+^CD16^−^ classical monocytes (sCD40L at week 2), CD20^+^ B cells (IL-18 at week 7), and NKG2a^+^ NK cells (IL-12/23 at week 7), while the only positive association in p12KO cells was between CD4^+^CD95^−^ naïve T cells and IL-6 (week 2). Negative associations, mostly observed early in WT-infected animals, were found with monocytes (IL-12/23 at week 2 and IL-6 at week 5), CD14^+^CD16^+^ intermediate monocytes (sCD40L at week 2 and MIP-1β at week 5), and CD4^+^CD95^−^ naïve T cells (IL-8 at week 5). Associations between cytokines and neutrophils (IL-18 at week 16) and CD8^+^ T cells (IL-8 at week 21) were observed at later timepoints. In contrast, p12KO animals displayed more negative cytokine/cell subset associations with CD14^−^CD16^+^ nonclassical monocytes (IL-1β at week 16 and IFN-γ at week 21) and CD4^+^ T cells (MIP-1α at week 21) at a later time, whereas the association of CD4^+^CCR4^+^ cells with IL-1β was found early, at week 5. In summary, the plasma levels of cytokines clearly indicate that the immune response induced by WT infection differs from that induced by the p12KO virus.

To gain more insight into the role of monocytes in restricting HTLV-1 infection, we evaluated changes in cytokine levels over time in triple-depleted compared with double-depleted animals [52] in each virus group (Figure 4B,C; Table S4). The alluvial plot comparing cytokine changes relative to baseline in the plasma of triple-depleted vs. double-depleted animals prior to infection with the WT virus shows significantly elevated IL-10 levels in the triple-depleted group only (pink, “↑ triple only”) at the assayed timepoints of the two studies (Figure 4B). While the cytokine levels fluctuated in both groups, more cytokines had elevated levels compared with baseline throughout the study in triple-depleted compared with double-depleted animals. In the double-depleted group, the IL-8 levels were elevated compared with baseline at weeks 2, 5, 8, and 21. At week 21, IL-1β was the only cytokine elevated in both triple- and double-depleted animals (Figure 4B).

The cytokine signature also differed between triple- and double-depletion groups in animals infected with HTLV-1_p12KO_, suggesting a difference in the immune response to the *orf-1*-knockout virus. A comparison of the changes in the cytokine levels in the plasma of the HTLV-1 _p12KO_-infected groups indicated that there was almost no overlap in the cytokine profile between the triple- and double-depleted groups (Figure 4C). Notably, in the HTLV_p12KO_-infected animals treated with either M-T807R1/Clodrosome (triple) or M-T807R1 alone (double), no cytokines had increased levels relative to baseline for both groups (Figure 4C and Table S4). The only cytokine increased in triple-depleted-HTLV-1_p12KO_ was IL-10 at week 21 (Figure 4C). In contrast, cytokines IL-8, IL-1β, and IL-18 increased at various timepoints in the study in double-depleted animals (Figure 4C). Surprisingly, the majority of cytokines remained the same or decreased compared with baseline levels in HTLV-1_p12KO_-infected animals until week 21. Together, these results suggest that monocyte depletion, with the concurrent absence of CD8^+^ and NK cells, increases the pro-inflammatory response in animals infected with HTLV-1_WT_, while infection with HTLV-1_p12KO_, regardless of the type of treatment, elicits a delayed and weaker plasma cytokine response.

### 3.4. Repeated Triple Depletion in Chronically HTLV-1-Infected Macaques Increased Antibody Responses but Not Viral DNA Levels

**Figure 5:**
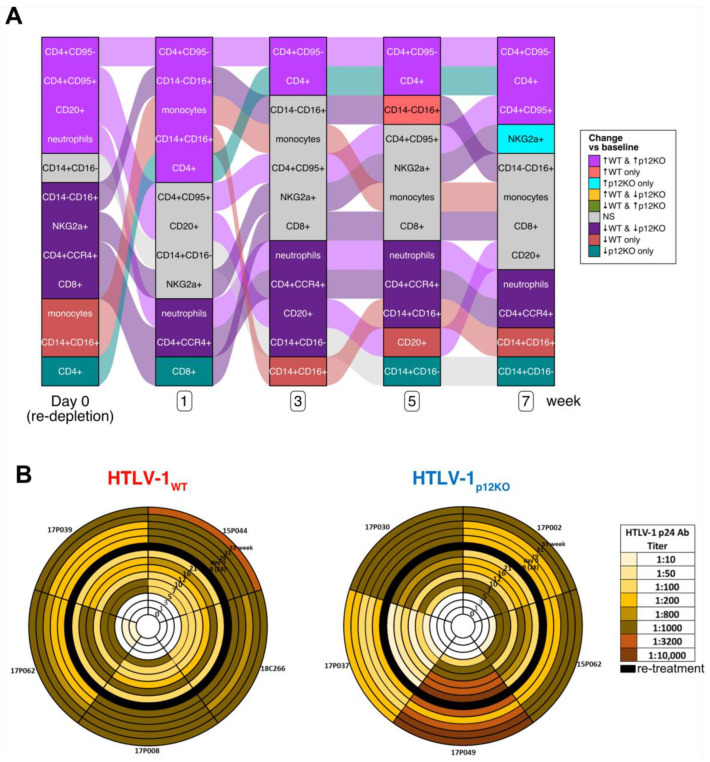
Cell populations and sero-reactivity in HTLV-1_WT_- and HTLV-1_p12KO_-infected macaques after re-depletion with M-T807R1/Clodrosome. (**A**) Alluvial plot illustrating the changes in CD8^+^ and CD4^+^ T-cell, B-cell (CD20^+^), and monocyte cell counts and the frequency of the three monocyte subsets (classical (CD14^+^CD16^−^), intermediate (CD14^+^CD16^+^) and non-classical (CD14^−^CD16^+^)), as well as NKG2a^+^ cells and neutrophils from whole blood of HTLV-1_WT_ (WT) and HTLV-1_p12KO_ (p12KO) infected animals, given for day 0 (re-depletion) and weeks 1, 3, 5, and 7 after re-treatment with M-T807R1/Clodrosome. Paired analysis fitting generalized estimating equations (geeGLM) determined that the cell populations significantly changed over time in each group. Statistical results are represented by ↑ for increase (*p* < 0.05), ↓ for decrease (*p* < 0.05), and NS for not significant (*p* ≥ 0.05). Each column is sorted according to the pattern of significant changes over time across the groups indicated. These patterns are represented by colors as described (legend at right of the plot). Streams (alluvia) between columns highlight the significance/direction pattern over time for each cell type and are colored according to the pattern on day 0 (re-depletion) vs. baseline (leftmost column). Timepoints and groups are shown below the graph. (**B**) The HTLV-1 p24Gag antibody titer was measured in macaques belonging to the HTLV-1_WT_ (left) and HTLV-1_p12KO_ (right) groups at weeks 1, 3, 5, 7, 10, 12, 16, and 21 and after re-treatment at week 28 (baseline, B); day 0; and weeks 29, 31, and 33. Black circles represent the starting point of re-treatment with M-T807R1/Clodrosome. Dilutions of 1:10, 1:50, 1:100, 1:200, 1:800, 1:1000, 1:3200, and 1:10,000 were used and color-coded as reported in the figure.

Because triple depletion enhanced infection, which was sustained for 28 weeks, the study was expanded with the hypothesis that re-depletion could recapitulate acute infection and increase both viral DNA and virus-specific immune responses. At week 28, infected macaques were treated again with M-T807R1/Clodrosome (Figure S4). Similar to previous observations, the treatment of macaques with M-T807R1/Clodrosome prior to infection resulted in NK and CD8^+^ cells becoming undetectable (day 0) in both the HTLV-1_WT_ and HTLV-1_p12KO_ groups (Figure S4). In addition, a marginal or null decrease in the percent of circulating monocytes was found (Figure S4). However, unlike what was seen above for the first triple depletion of uninfected macaques, when infected macaques were treated with M-T807R1/Clodrosome, the percent of NK and CD8^+^ cells began to recover 1 week after depletion in HTLV-1_WT_-infected animals and 3 weeks after depletion in HTLV-1_p12KO_-infected animals (Figure S4). An alluvial plot comparing changes in cell populations over time after re-depletion in both virus groups indicates a transient increase in monocytes at week 1, which returned to day 0 (re-depletion) levels by week 3, in macaques infected with both viruses (Figure 5A). Interestingly, after re-depletion, a significant decrease in neutrophils was reported from week 1 to the end of the study in both groups, which is in contrast to the increased neutrophil levels after M-T807R1/Clodrosome treatment in uninfected animals (Figure 1, Figure 5A and Figure S4). We observed a sustained decrease in the percentage of CD4^+^CCR4^+^ cells from re-depletion (day 0) up to week 35 (7 weeks after re-depletion; Figure 5A and Figure S4). This was unexpected, as CD4^+^CCR4^+^ cells are thought to be preferentially infected by HTLV-1 [58,59,60,61].

Upon re-depletion of cell subsets in the infected macaques, the serum p24Gag antibody titers increased in eight of the ten macaques (Figure 5B). The two animals which maintained or had reduced p24Gag titers were the HTLV-1_p12KO_-infected animals, 17P030 and 17P049, that had high p24 titers prior to re-treatment (1:1000 and 1:10,000, respectively; Figure 5B). Re-depletion did not increase viral DNA levels in the blood in either HTLV-1_WT_- or HTLV-1 _p12KO_-infected animals (Figure S5A,B). Similarly, the frequency of detection of the *gag* and *orf-1* genes by nested PCR in the blood did not increase after re-depletion (Figure S5C,D). Not surprisingly, the mRNA expression of *tax* and *hbz* was not detected in PBMCs throughout the study or after re-depletion. The increase in antibody titers against p24Gag without an obvious decrease in blood viral DNA likely reflects a subliminal increase in viral replication in tissues.

### 3.5. Cytokine Profile in Plasma after Repeated Treatment with M-T807R1/Clodrosome in Infected Macaques

**Figure 6:**
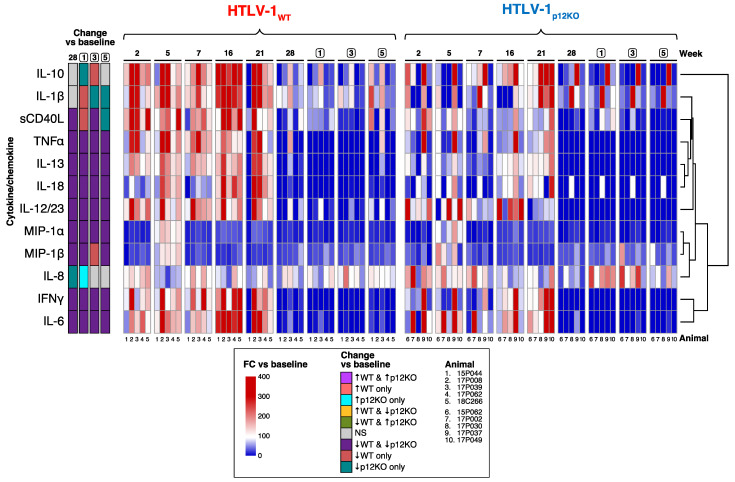
Comparison of cytokine profiles of peripheral whole blood of animals infected with HTLV-1_WT_ or HTLV-1_p12KO_ after first and second triple depletion of NK cells, CD8^+^ cells, and monocytes. The cytokine levels were analyzed by using the MILLIPLEX MAP Non-Human Primate Cytokine Magnetic Bead Panel kit (Millipore Sigma). Changes over time for each variable were computed with generalized estimating equations with animal ID as a random effect. For visualization, the FC for each animal was calculated as 100 * (week XX + 0.0001)/(baseline + 0.0001) and graphed as a heatmap, where increases are shown in red and decreases in blue. The scale is given under the plot along with the animal IDs. Statistical information is shown in the first 5 columns on the left of the heatmap and is color-coded, indicating which cytokines changed over time vs. baseline after the second triple depletion (*p* < 0.05). The color code (↑ for increase and ↓ for decrease) is given under the heatmap. Gray indicates a non-significant change (*p* > 0.05). The group name and timepoints are indicated above the heatmap, the cytokine/chemokine names on the left of the plot, and animal numbers below the graph.

To assess the differences in immunological response after the first and second triple depletion, the cytokine profiles were compared in each tested group. A significant reduction in most cytokine levels was measured at week 28 after infection relative to baseline (start of the experiment) for both the HTLV-1_WT_ and HTLV-1p12KO groups. Only IL-10 and IL-1β were unchanged at week 28 relative to baseline levels (Figure 6). Although the re-depletion of NK cells, CD8^+^ cells, and monocytes in both the WT and p12KO groups caused a sustained increase in p24Gag antibody titers, most cytokine levels remained below baseline levels (Figure 6). There were some notable differences between the two virus groups in IL-10, IL-8, IL-1β, and sCD40L levels. IL-1β decreased below baseline levels early (week 1 post-re-depletion) in the HTLV-1_WT_-infected animals and with a delay (weeks 3 and 5 post-re-depletion) in the HTLV-1_p12KO_-infected animals (Figure 6). In contrast, IL-10 levels were lower than at baseline at week 1 post-re-depletion and at week 3 in the HTLV-1_p12KO_- and HTLV-1_WT_-infected animals, respectively (Figure 6). Interestingly, IL-8 remained at baseline levels in the HTLV-1_WT_ group but increased above baseline levels at 1 week in the HTLV-1_p12KO_ animals (Figure 6). Similar to what was found for the first triple depletion, significant correlations between changes in cytokine levels and changes in cell counts/frequencies after re-depletion were sporadic in both virus groups (Figure S6).

At the end of the study, we examined virus dissemination to various tissues by using nested PCR for the *gag* and *orf-I* genes in all animals triple-depleted twice and compared the results with the animals that had been subjected to the double depletion of NK and CD8^+^ cells. We found no difference in the pattern of viral DNA distribution in the two virus groups regardless of the depleting drug treatment or frequency (Figure S7A). No reversion of the single nucleotide mutation in HTLV-1_p12KO_ was observed in animals after re-treatment with M-T807R1/Clodrosome.

**Figure 7:**
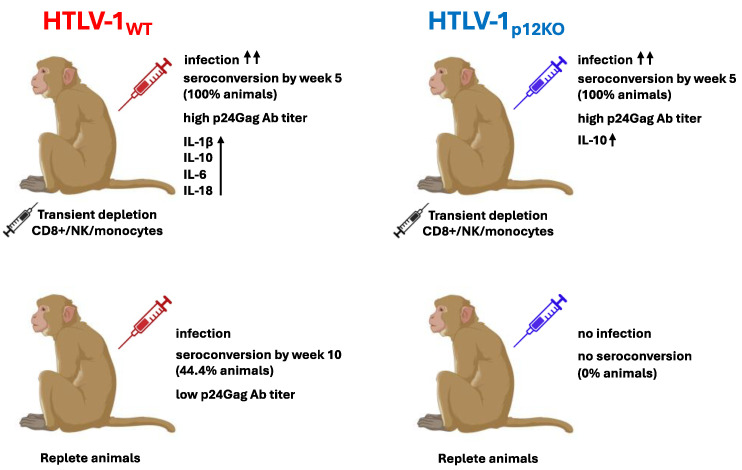
Model of HTLV-1 infection in macaques. A diagram illustrating virus–host responses in transiently triple-depleted (top) or replete (bottom) animals is shown. Treatment of macaques with M-T807R1/Clodrosome (black syringe) to deplete CD8+, NK cells, and monocytes prior to intravenous infection with HTLV-1_WT_ (red) or HTLV-1_p12KO_ (blue) resulting in seroconversion, detection of PVL, and high p24Gag antibody titers in 10 out of 10 animals. Completely restoring HTLV-1_p12KO_ infection. While viral parameters were similar in the two groups, a difference in cytokine/chemokine profiles was measured. In contrast, in untreated/repleted animals, the HTLV-1_p12KO_ virus could not sustain infection, with no seroconversion or detection of PVL. HTLV-1_WT_ infection occurred in 44% of repleted animals, with delayed seroconversion and low p24Gag antibody titers. The ↑↑ indicates increased infectivity (100%). The ↑ signifies high plasma levels of indicated cytokine.

Taken together, these results indicate that in addition to CD8^+^ and NK cells, early engagement of monocytes/macrophages in HTLV-1 infection contributes to anti-viral immunity (see Figure 7). Triple depletion prior to infections results in a more acute infection with consistently measurable viral DNA levels. In addition, triple depletion prior to infection with HTLV-1_p12KO_ completely restores infection, mimicking the viral parameters of HTLV-1_WT_, but with differing cellular responses.

## 4. Discussion

Typical host responses to virus infection follow a pattern of early induction of type 1 interferon from dendritic cells, increased IL-15 release, and NK cell and B-cell engagement, resulting in the release of cytokines/chemokines that drive a strong Th1-type adaptive response, followed by the proliferation of T lymphocytes and the production of antibodies impacting viral replication, clearance, or persistence [62]. Persistent HTLV-1A infection is linked to its ability to escape host immunity, in part via Orf-1 regulatory protein functions, which counteract CTL and NK cell killing. Orf-1, although dispensable for virus replication in vitro, is necessary for sustained infection in rhesus macaques, and sequence analyses suggest that Orf-1 expression is maintained in the majority of HTLV-1-infected individuals [23,24,47,51,63]. In addition, depletion of NK and CD8^+^ cells in the rhesus macaque model enhanced HTLV-1A infection and allowed HTLV-1_p12KO_, virus not expressing the viral *orf-1* gene, to establish infection [52]. Consistently with the importance of NK and CTL responses in modulating HTLV-1 infection, when chronically HTLV-infected macaques were treated with the immunomodulatory drug pomalidomide, a transient increase in the surface expression of B7-2, CD11b, HLA-DR, and CD69 was measured, as well as an increase in proliferating CD4^+^, CD8^+^, and NK cells. Unfortunately, pomalidomide treatment also caused the activation of HTLV-1 in infected macaques, limiting its usefulness in the treatment of HTLV-1 infection [64]. The importance of NK and CD8^+^ cells in controlling HTLV-1A was also demonstrated in the cynomolgus macaque model [65]. Although the depletion of monocytes/macrophages did not fully restore the infectivity of the *orf-1*-knockout virus, monocyte/macrophage depletion did enhance the seroconversion of wild type HTLV-1A [52]. Because of the complex interplay among NK cells, CD8^+^ cells, and monocytes/macrophages in regulating the immune response to infection and vaccine efficacy, it is important to understand the role that each plays in determining the quality of the host immune response to HTLV-1 infection.

In the current study, we found that treatment with M-T807R1/Clodrosome aimed at simultaneously depleting monocytes, NK cells, and CD8^+^ cells prior to infection ultimately did not shorten the time to seroconversion or the induction of p24Gag antibody titers of HTLV-1_WT_, but it did accelerate seroconversion in macaques exposed to HTLV-1_p12KO_ when compared with treatment with M-T807R1 alone and resulted in more consistent detection of viral DNA in the two virus groups (Figure 4; [52]). The timing of seroconversion in macaques exposed to the virus in this study falls within the range of what has been reported for a human cohort of transfusion recipients exposed to HTLV-1 who developed antibodies against core, envelope, and tax proteins 4–8 weeks following primary infection [66]. Data from the human patients indicated that the predominant immune response is to p24Gag, and antibodies against the p21 envelope protein frequently appear before those to the gp46 envelope protein or one of the latest responses to the Tax protein [66]. In our study, the breadth and magnitude of the response also seem to be similar. Neutralizing antibodies against the HTLV-1 envelope are detected in the plasma of infected individuals, and passive immunization protected cynomolgus macaques from infection and prevented viral transmission in NOD-SCID/γc-null mice and rabbits [67,68,69]. In addition, a correlation between anti-HTLV-1 antibodies at birth in infants born to HTLV-1-infected mothers inversely correlated with risk of infection [70]. Thus, in the rhesus macaque model, triple depletion prior to infection better mimics human infection via blood products. It will be interesting to further characterize the functional antibody responses we have elicited in this animal model.

Alterations in immune responses are associated with HTLV-1 infection and contribute to disease development [10,27,28,71,72,73,74]. While CD4 cells are the primary target of HTLV-1, the virus is also found in CD8, monocytes, macrophages, and dendritic cells from infected individuals [75,76,77,78,79,80]. Viral infection significantly alters T-cell and dendritic cell functions, increases the frequency of intermediate and non-classical monocytes (pro-inflammatory monocytes), and decreases the frequency of classical monocytes that mediate apoptotic cell clearance and maintain tissue homeostasis [79,81,82]. The continuous but ineffective attempts of the immune system to clear HTLV-1 may result in chronic inflammation with exhaustion of both NK and CD8 cells, as reported in infected individuals with high virus burden [74,83,84,85,86,87,88,89]. Although, in the current study, the depletion of monocytes in the blood was minimal, it is possible that tissue macrophage depletion was more pronounced, as has been reported [90,91]. Indeed, we determined that simultaneously depleting monocytes/macrophages with NK and CD8 cells prior to infection with either the WT or p12KO virus results in a more robust infection than depleting either NK or CD8^+^ cells alone.

HTLV-1 infection is associated with a disruption of cytokine homeostasis and the balance between inflammatory and anti-inflammatory responses. In HAM/TSP patients, there is increased production of pro-inflammatory cytokines such as IFN-γ, TNFα, IL-1β, and IL-16, and the neurotoxic cytokines IFN-γ and TNFα are found in high concentrations in the spinal fluid [92,93,94]. Individuals with ATLL are reported to have high levels of IL-10 and TGFβ, which may contribute to immunosuppression [94]. In the triple-depletion model presented here, we found that treatment with M-T807R1/Clodrosome prior to infection resulted in an augmented inflammatory signature, as indicated by increased plasma IL-1β, IFN-γ, IL-10, IL-6, and IL-18 levels in the WT-infected animals. We observed a distinct difference in IL-10 expression, where IL-10 increased at all timepoints relative to baseline in HTLV-1_WT_-infected animals but only week 21 in HTLV-1_p12KO_-infected animals. In addition, repeated treatment with depleting drugs transiently induced higher IL-8 plasma levels only in the HTLV-1_WT_-infected animals. We did detect a decrease in CCR4^+^CD4^+^ memory T cells at week 21 after infection in both the HTLV-1_WT_- and HTL-1_p12KO_-infected animals even after the re-depletion of NK cells, CD8^+^ cells, and monocytes. As the overall frequencies of CD4^+^ cells were at baseline levels or higher in both groups, this may indicate the trafficking of CCR4+CD4+ cells to tissue compartments or the proliferation of a CCR4^−^CD4^+^ cell population. Further characterization of CD4^+^ cells will be helpful to determine specific frequencies of Th1, Th2, TH17, and Treg cell populations over time in this animal model and their role in the response to viral infection [95,96]. In contrast, after infection, we observed higher frequencies of neutrophils and monocyte subsets, cells known to synthesize pro-inflammatory cytokines that contribute to the host response to viral disease but that can also contribute to tissue damage [97,98].

The current study, while confirming prior results underscoring the key role of NK cells in acute HTLV-1 infection [52], also highlights the role of monocytes/macrophages in early infection. In HIV, monocytes/macrophages have been shown to contribute to persistence by acting as a viral reservoir and transmitting virus [99]. While we have shown that the three monocyte subsets carry HTLV-1 DNA in infected individuals [79], our current and previous [52] results suggest that the early engagement of monocytes greatly contributes to anti-viral immunity. Monocytes are among the first lines of defense against viral infection. Monocytes/macrophages present antigens, have phagocytic activity, release reactive oxygen species, produce cytokines/chemokines, and modulate T-cell responses [98,100]. Our recent work showed that in addition to evading NK and CTL responses, the HTLV-1 Orf-1 protein is associated with influencing inflammasome activation and decreasing monocyte engulfment [24,52]. Our current work indicates that Orf-1 plays additional roles in host immunity, as HTLV-1_p12KO_ infectivity in macaques is rescued to the level observed with HTLV-1_WT_ following the depletion of NK cells, CD8^+^ cells, and monocytes, which was not observed with the depletion of both NK and CD8^+^ cells. The distinct cellular and cytokine profiles measured during the establishment of infection by HTLV-1_WT_ and HTLV-1_P12KO_ may influence viral pathogenicity and require further investigation.

## Data Availability

The original contributions presented in the study are included in the article/supplementary material, and further inquiries can be directed to the corresponding author(s).

## Supplementary Materials

The following supporting information can be downloaded at: https://www.mdpi.com/article/10.3390/pathogens13040292/s1, Figure S1: Gating strategy, Figure S2: Changes in immunophenotypic profiles of whole peripheral blood of animals infected with HTLV-1_WT_ or HTLV-1_p12KO_, Figure S3: Associations between the changes in frequency of cells and changes in cytokine/chemokine levels over time, Figure S4: HTLV-1_WT_ and HTLV-1_p12KO_ infectivity after re-depletion using M-T807R1/Clodrosome, Figure S5: Viral DNA in PBMCs of HTLV-1_WT_- and HTLV-1_p12KO_-infected macaques after re-depletion, Figure S6: Associations between the changes in frequency of cells and changes in cytokine/chemokine levels after re-depletion with M-T807R1/Clodrosome, Figure S7: Viral dissemination in HTLV-1-infected animals treated with M-T807R1 or M-T807R1/Clodrosome prior to infection, Table S1: Animals’ prior history, Table S2: Antibodies used for non-human primate flow cytometry, Table S3: Cell frequencies over time, Table S4: Changes in cytokine/chemokine levels.
