# Supplementary material for: Complete Rescue of HTLV-1p12KO Infectivity by Depletion of Monocytes Together with NK and CD8+ T Cells
Source: Pathogens. 2024 Mar 29;13(4):292. doi: 10.3390/pathogens13040292 (PMC11054408; doi:10.3390/pathogens13040292)
Supplement: Supplementary file 1 [file pathogens-13-00292-s001.zip › Supplementary Figures 2-23-24.pdf]

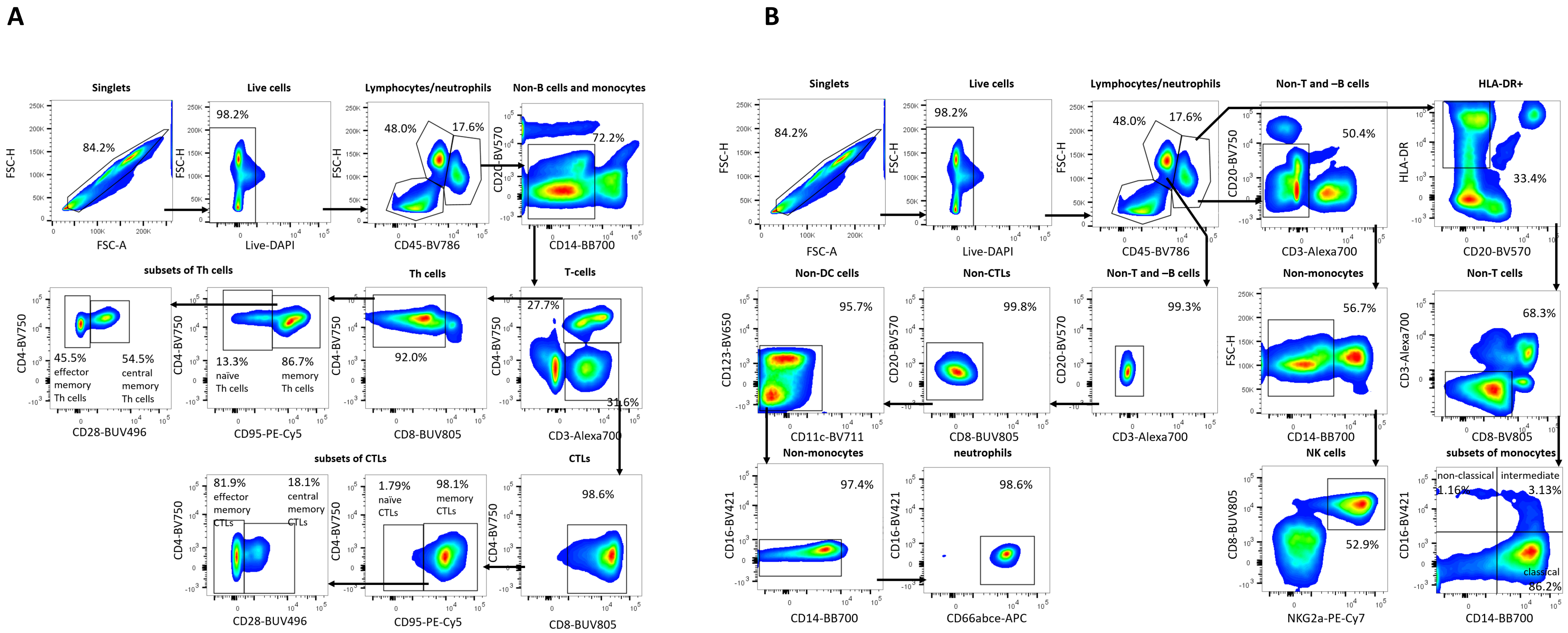
A
Singlets
84.2%
FSC-H
FSC-A
Live cells
98.2%
Live-DAPI
Lymphocytes/neutrophils
48.0%
17.6%
CD45-BV786
Non-B cells and monocytes
72.2%
CD20-BV570
CD14-BB700
subsets of Th cells
45.5% effector memory Th cells
54.5% central memory Th cells
CD4-BV750
CD28-BUV496
13.3% naïve Th cells
86.7% memory Th cells
CD95-PE-Cy5
Th cells
92.0%
CD8-BUV805
T-cells
27.7%
31.6%
CD3-Alexa700
subsets of CTLs
81.9% effector memory CTLs
18.1% central memory CTLs
1.79% naïve CTLs
98.1% memory CTLs
CTLs
98.6%
B
Non-T and –B cells
50.4%
CD20-BV750
HLA-DR+
33.4%
HLA-DR
CD20-BV570
Non-DC cells
95.7%
CD123-BV650
CD11c-BV711
Non-CTLs
99.8%
Non-T and –B cells
99.3%
Non-monocytes
56.7%
FSC-H
CD14-BB700
Non-T cells
68.3%
CD3-Alexa700
CD8-BV805
Non-monocytes
97.4%
CD16-BV421
neutrophils
98.6%
CD66abce-APC
NK cells
52.9%
CD8-BUV805
NKG2a-PE-Cy7
subsets of monocytes
non-classical 1.16%
intermediate 3.13%
classical 86.2%

Figure S1

A

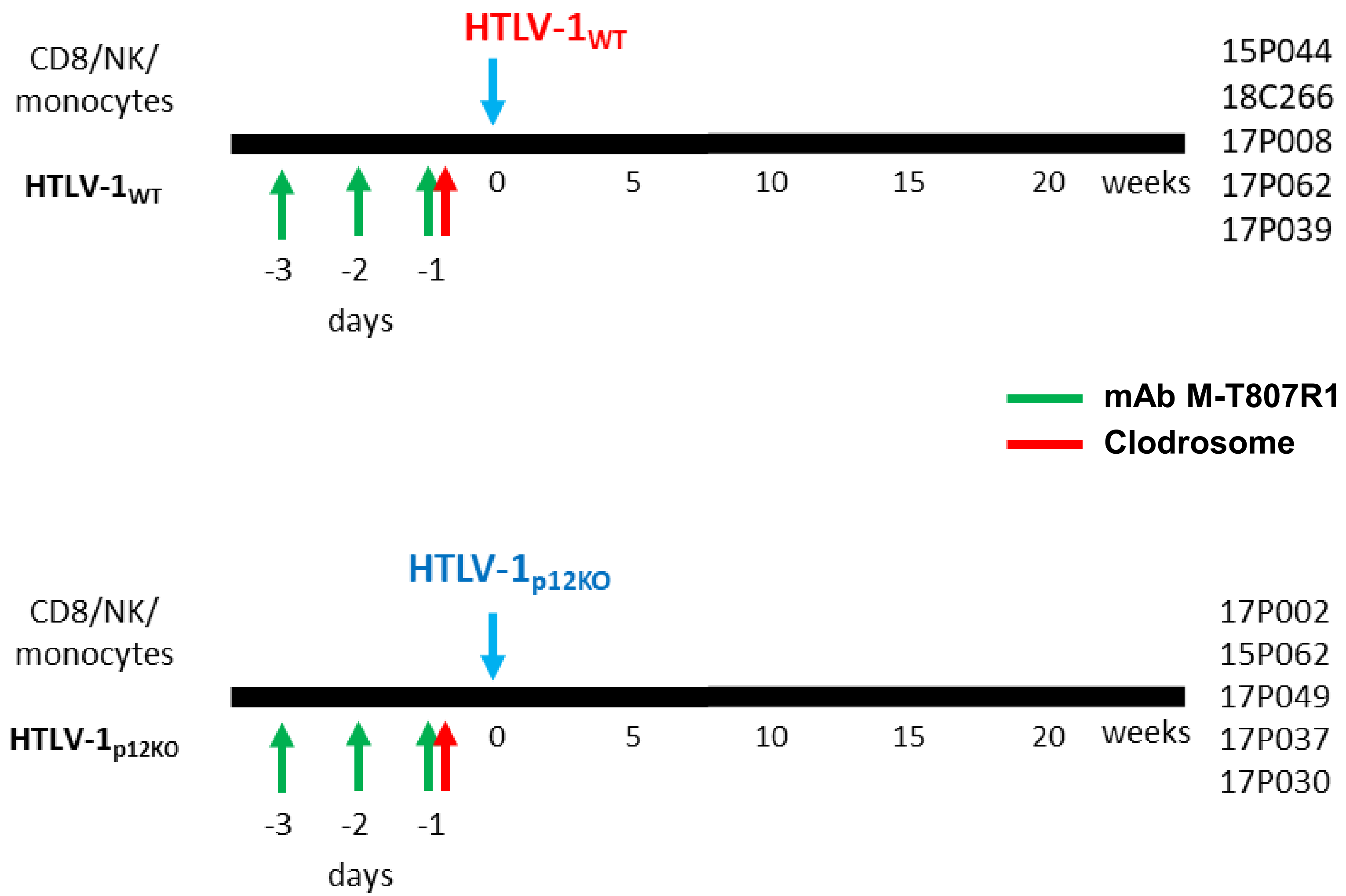

HTLV-1WT
CD8/NK/
monocytes
HTLV-1WT
0
5
10
15
20
weeks
-3
-2
-1
days
15P044
18C266
17P008
17P062
17P039
mAb M-T807R1
Clodrosome
HTLV-1p12KO
CD8/NK/
monocytes
HTLV-1p12KO
0
5
10
15
20
weeks
-3
-2
-1
days
17P002
15P062
17P049
17P037
17P030

Figure S2

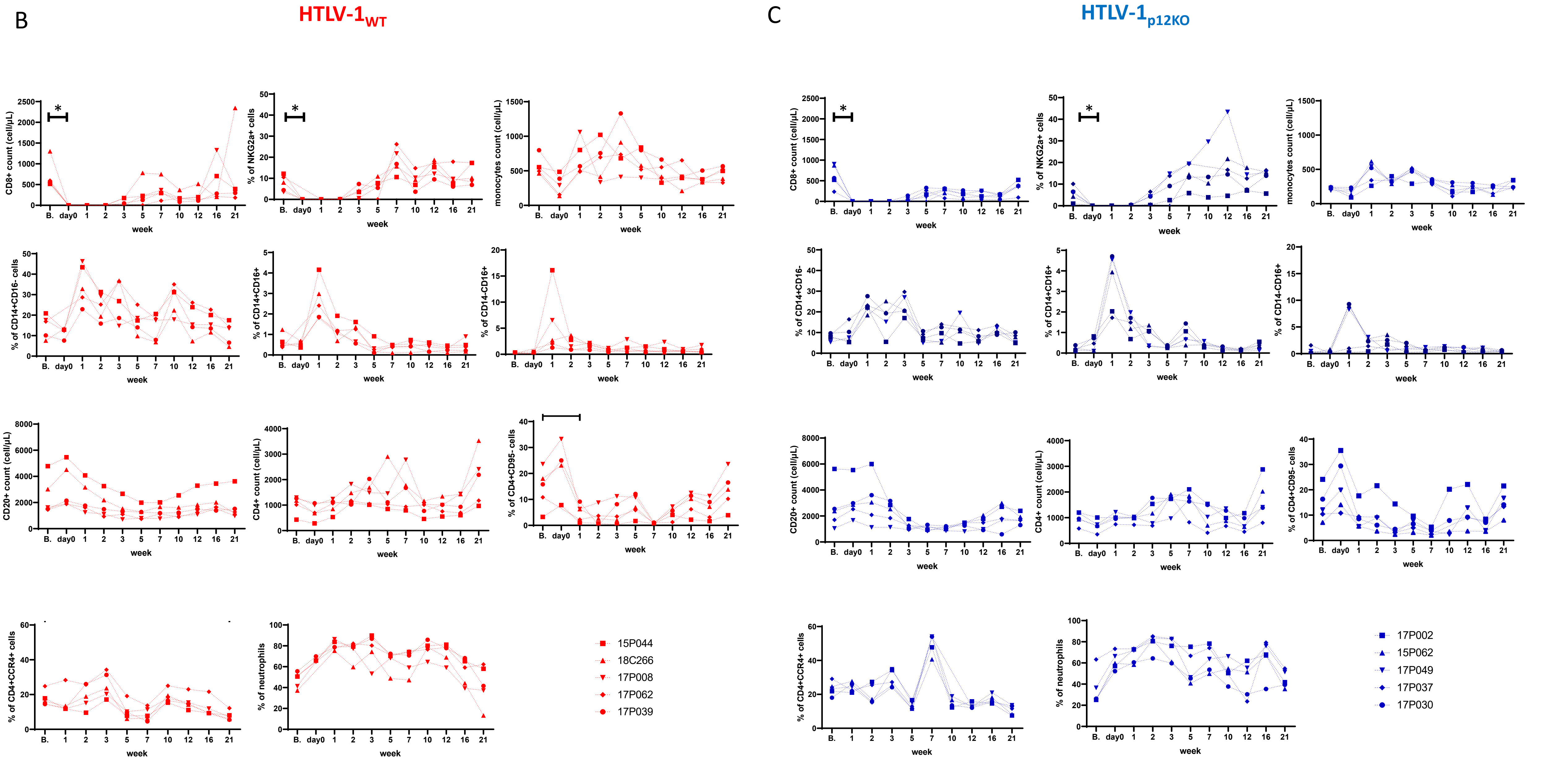
B
HTLV-1WT
C
HTLV-1p12KO
CD8+ count (cell/µL)
% of NKG2a+ cells
monocytes count (cell/µL)
% of CD14+CD16- cells
% of CD14+CD16+
% of CD14-CD16+
CD20+ count (cell/µL)
CD4+ count (cell/µL)
% of CD4+CD95- cells
% of CD4+CCR4+ cells
% of neutrophils
week
B.
day0
15P044
18C266
17P008
17P062
17P039
17P002
15P062
17P049
17P037
17P030

Figure S2

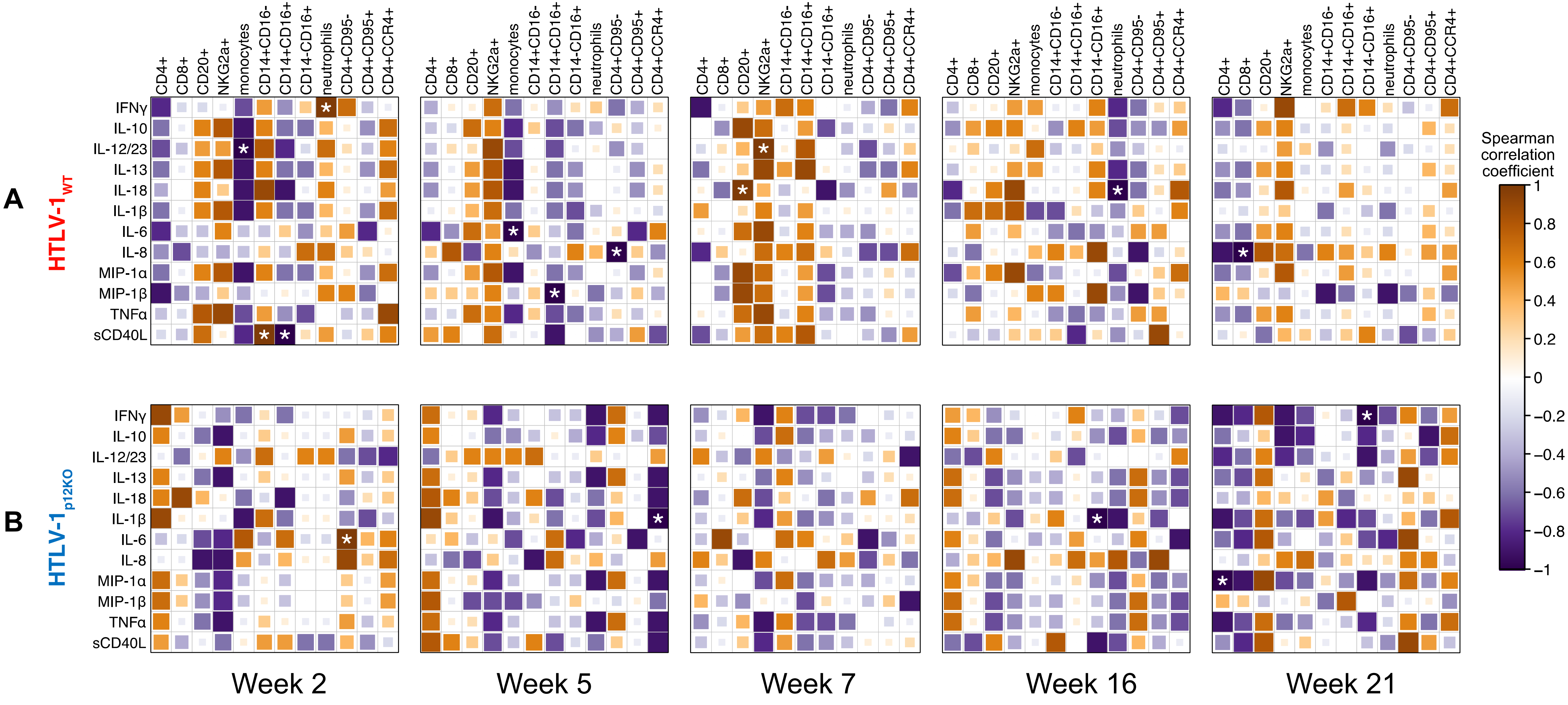
A
HTLV-1WT
B
HTLV-1p12KO
CD4+
CD8+
CD20+
NKG2a+
monocytes
CD14+CD16-
CD14+CD16+
CD14-CD16+
neutrophils
CD4+CD95-
CD4+CD95+
CD4+CCR4+
IFNγ
IL-10
IL-12/23
IL-13
IL-18
IL-1β
IL-6
IL-8
MIP-1α
MIP-1β
TNFα
sCD40L
Week 2
Week 5
Week 7
Week 16
Week 21
Spearman correlation coefficient
1
0.8
0.6
0.4
0.2
0
−0.2
−0.4
−0.6
−0.8
−1

Figure S3

C

first triple depletion	HTLV-1$_{WT}$ cell population	HTLV-1$_{WT}$ cytokine	HTLV-1$_{WT}$ time interval vs baseline	HTLV-1$_{p12KO}$ cell population	HTLV-1$_{p12KO}$ cytokine	HTLV-1$_{p12KO}$ time interval vs baseline
positive: ↑ cell population ↑ cytokine	CD14+CD16-	sCD40L	week 2	CD4+CD95-	IL-6	week 2
	neutrophils	IFNγ	week 2			
	NKG2a+	IL-12/23	week 7			
	CD20+	IL-18	week 7			
	monocytes	IL-12/23	week 2	CD4+CCR4+	IL-1β	week 5
negative: ↑ cell population ↓ cytokine	monocytes	IL-6	week 5	CD14+CD16+	IL-1β	week 16
	CD14+CD16+	sCD40L	week 2	CD14-CD16+	IFNγ	week 21
	CD14+CD16+	MIP-1β	week 5	CD4+	MIP-1α	week 21
	neutrophils	IL-18	week 16			
	CD4+CD95-	IL-8	week 5			
	CD8+	IL-8	week 21			

Figure S3

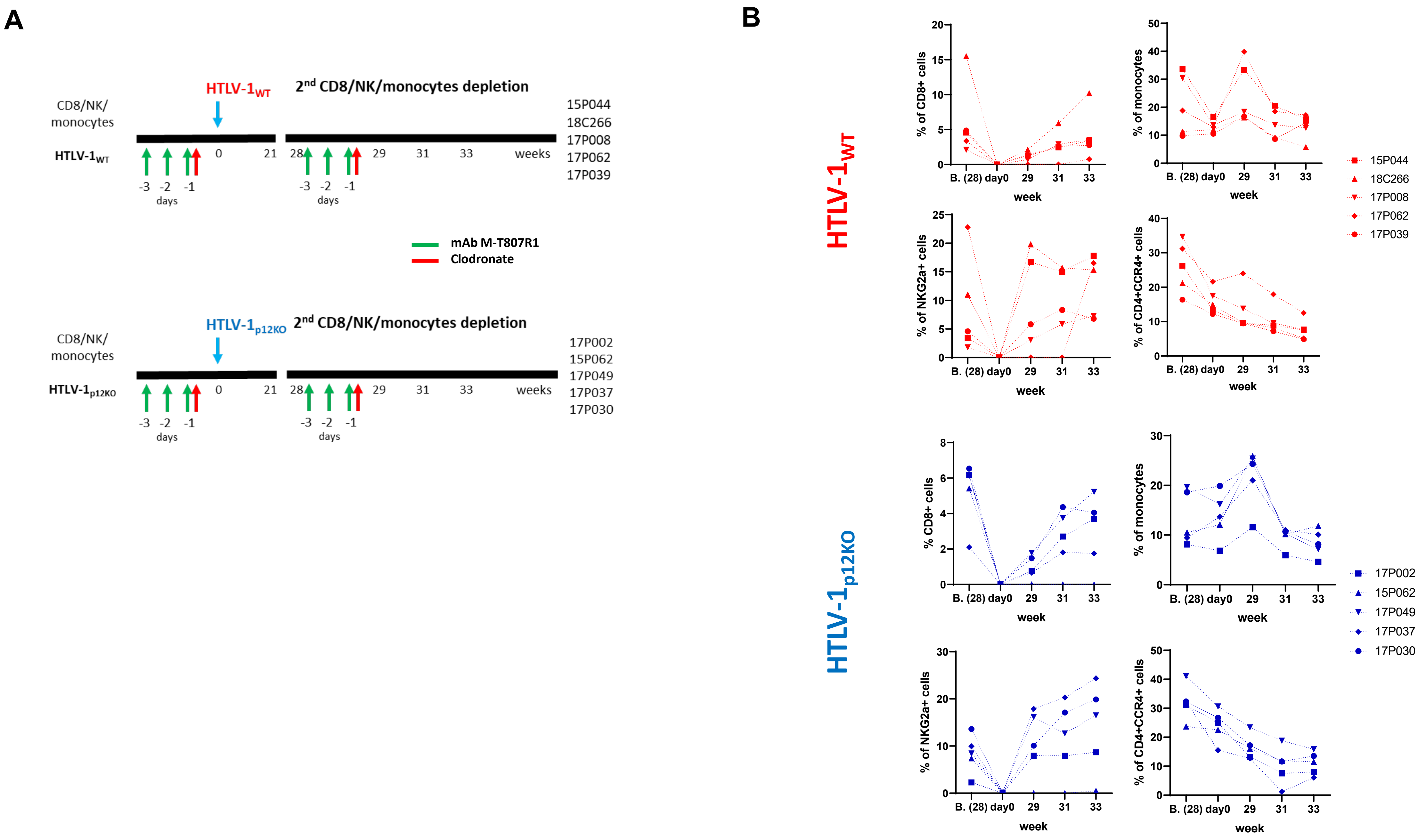
A
HTLV-1WT
2nd CD8/NK/monocytes depletion
CD8/NK/ monocytes
HTLV-1WT
0
21
28
29
31
33
weeks
-3 -2 -1
days
15P044
18C266
17P008
17P062
17P039
mAb M-T807R1
Clodronate
HTLV-1p12KO
2nd CD8/NK/monocytes depletion
CD8/NK/ monocytes
HTLV-1p12KO
17P002
15P062
17P049
17P037
17P030
B
HTLV-1WT
HTLV-1p12KO
% of CD8+ cells
% of monocytes
% of NKG2a+ cells
% of CD4+CCR4+ cells
% CD8+ cells
week
B. (28) day0 29 31 33

Figure S4

A

group	animal	PVL (%)				
		Baseline (28 week)	day 0	29 week	31 week	33 week
HTLV-1$_{WT}$	15P044	0.80	-	-	-	-
	18C266	-	0.19	-	0.23	0.04
	17P008	-	0.08	-	-	-
	17P062	-	0.14	0.21	-	-
	17P039	-	0.73	1.00	0.51	-

B

group	animal	PVL (%)				
		Baseline (28 week)	day 0	29 week	31 week	33 week
HTLV-1$_{p12KO}$	17P002	-	0.83	-	0.29	-
	15P062	0.10	0.26	0.58	-	-
	17P049	-	0.18	-	-	-
	17P037	0.17	-	-	-	-
	17P030	-	-	0.14	-	-

C

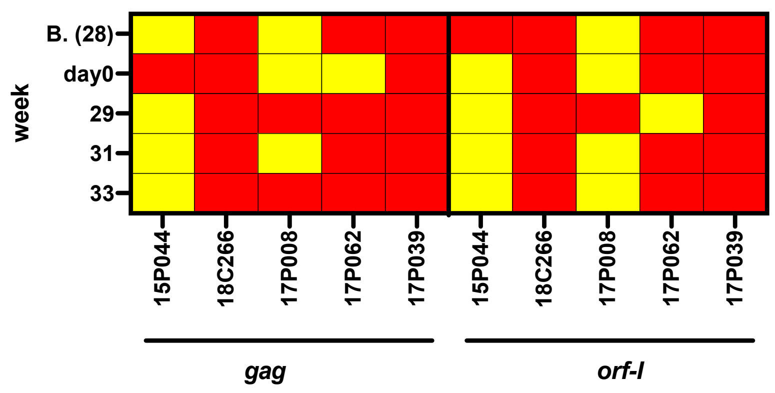

D

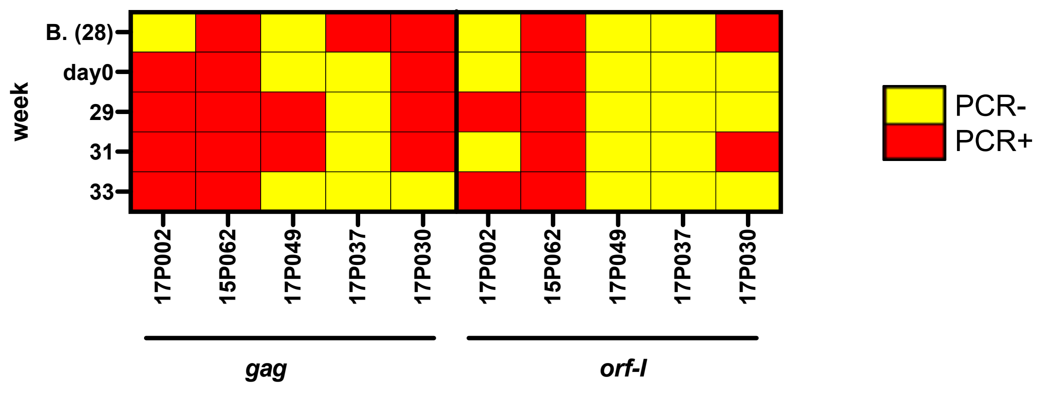

Figure S5

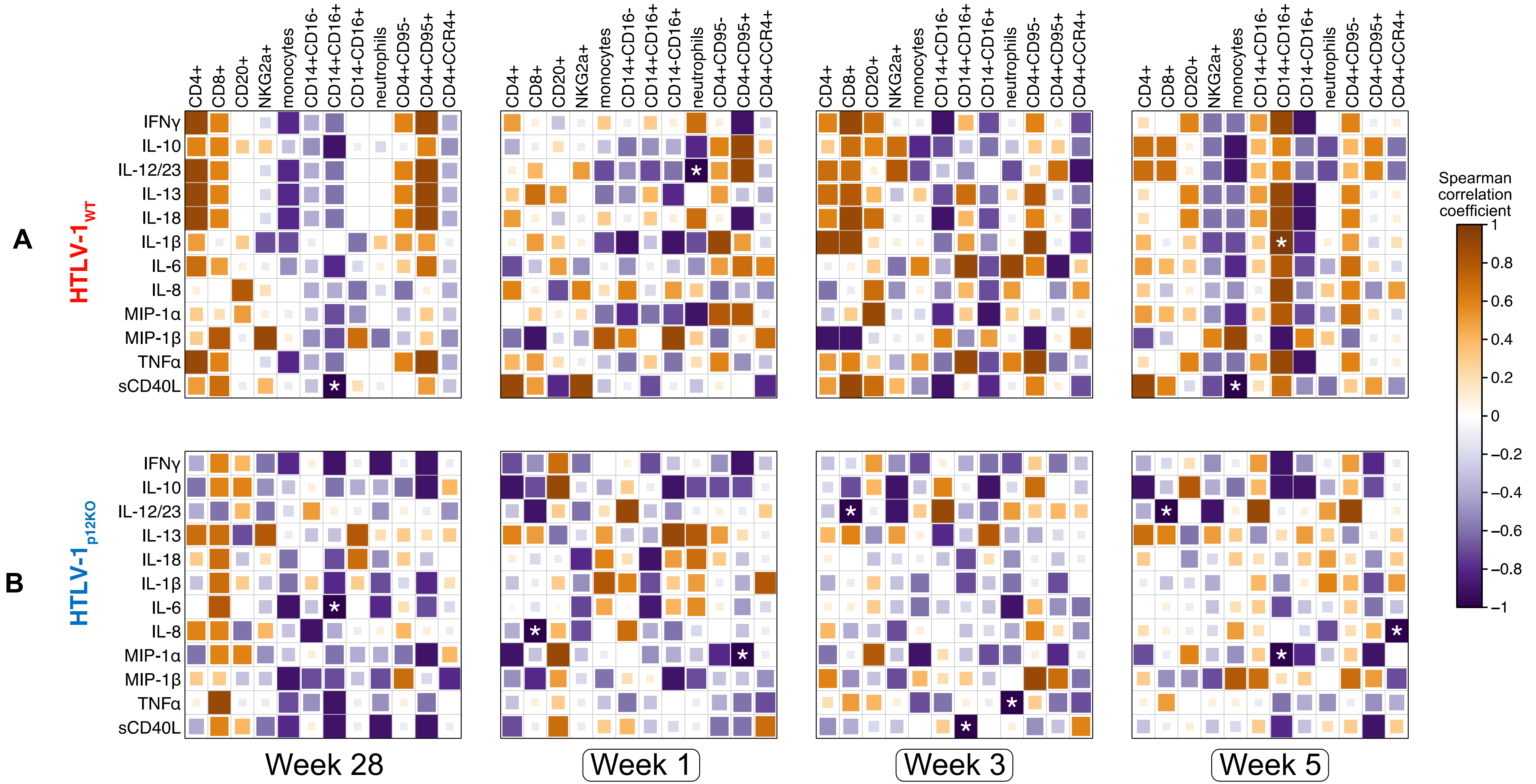
A
HTLV-1 WT
B
HTLV-1 p12KO
CD4+
CD8+
CD20+
NKG2a+
monocytes
CD14+CD16-
CD14+CD16+
CD14-CD16+
neutrophils
CD4+CD95-
CD4+CD95+
CD4+CCR4+
IFNγ
IL-10
IL-12/23
IL-13
IL-18
IL-1β
IL-6
IL-8
MIP-1α
MIP-1β
TNFα
sCD40L
Spearman correlation coefficient
1
0.8
0.6
0.4
0.2
0
−0.2
−0.4
−0.6
−0.8
−1
Week 28
Week 1
Week 3
Week 5

Figure S6

C

second triple depletion	HTLV-1$_{WT}$ cell population	HTLV-1$_{WT}$ cytokine	HTLV-1$_{WT}$ time interval vs baseline	HTLV-1$_{p12KO}$ cell population	HTLV-1$_{p12KO}$ cytokine	HTLV-1$_{p12KO}$ time interval vs baseline
positive: ↑ cell population ↑ cytokine	CD14+CD16+	IL-1β	week 5			
	CD14+CD16+	sCD40L	week 28	CD14+CD16+	IL-6	week 28
	monocytes	sCD40L	week 5	CD14+CD16+	sCD40L	week 3
negative: ↑ cell population ↓ cytokine	neutrophils	IL-12/23	week 1	CD14+CD16+	MIP-1α	week 5
				CD8+	IL-8	week 1
				CD8+	IL-12/23	week 3
				CD8+	IL-12/23	week 5
				CD4+CCR4+	IL-8	week 5
				CD4+CD95+	MIP-1α	week 1
				neutrophils	TNFα	week 3

Figure S6

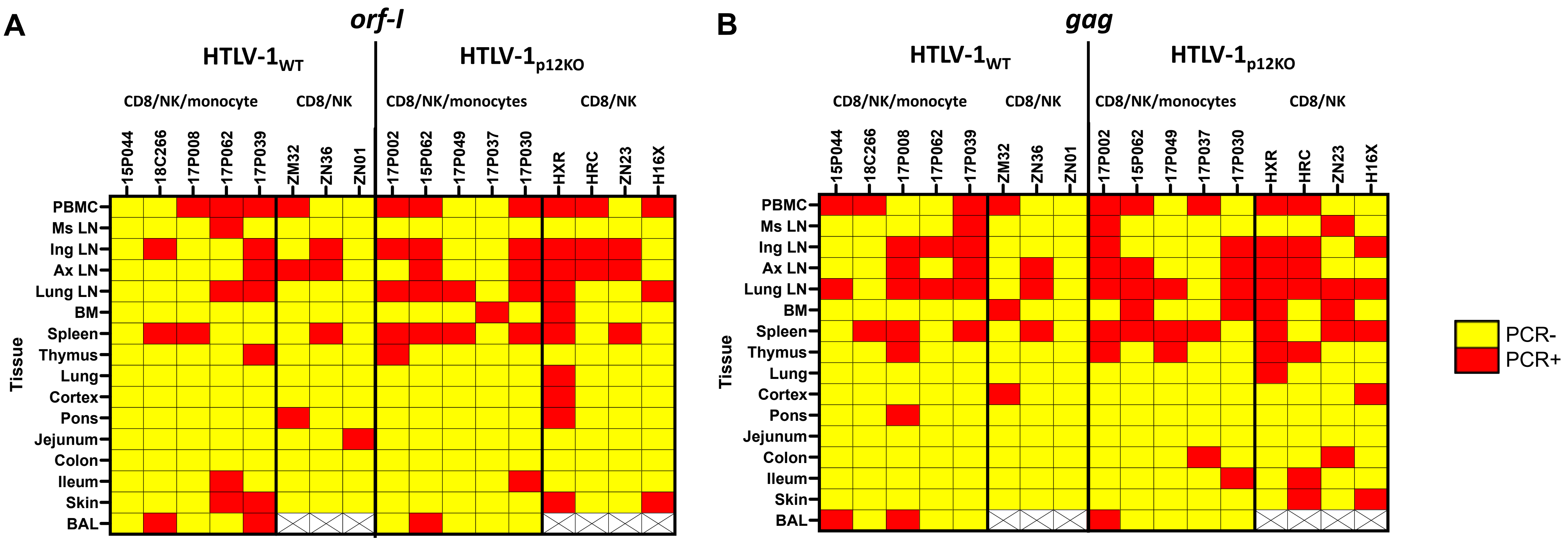

C

group	animal	PVL (%)															
		PBMC	BM	spleen	Ms LN	Ax LN	Ing LN	Lung LN	Cortex	Pons	Colon	Jejunum	Ileum	Thymus	Lung	Skin	BAL
WT	15P044	-	-	-	-	-	-	-	1.89	-	-	-	-	-	-	-	-
	18C266	-	0.44	-	-	-	-	-	-	-	-	-	-	-	-	-	0.12
	17P008	-	-	-	-	-	-	-	-	-	-	-	-	-	-	-	-
	17P062	-	0.44	-	-	-	-	0.07	-	-	-	-	-	-	-	-	-
	17P039	-	-	-	-	0.62	0.42	0.24	0.25	0.12	0.22	0.18	0.24	0.53	-	-	0.07
p12KO	17P002	0.01	-	0.04	-	0.01	-	-	-	-	-	-	-	-	0.3	-	-
	15P062	0.56	-	-	-	-	-	-	-	-	0.16	-	-	-	-	-	-
	17P049	-	-	-	-	-	-	-	-	-	-	-	-	0.08	-	-	0.71
	17P037	-	-	-	0.06	-	-	-	-	-	-	-	-	-	-	-	2.51
	17P030	-	-	-	0.06	0.16	0.23	0.06	-	-	0.04	0.1	-	-	-	-	-

Figure S7
