# Supplementary material for: Complete Rescue of HTLV-1p12KO Infectivity by Depletion of Monocytes Together with NK and CD8+ T Cells
Source: Pathogens. 2024 Mar 29;13(4):292. doi: 10.3390/pathogens13040292 (PMC11054408; doi:10.3390/pathogens13040292)
Supplement: Supplementary file 1 [file pathogens-13-00292-s001.zip › Supplementary Material 2-23-24.pdf]

Supplementary Material

Figure S1. Gating strategy. The gating strategy used for flow cytometry evaluation of the different (**A**) T-cell subsets**, (B**) neutrophils, NK cells, and monocyte subsets in whole peripheral blood.

Figure S2. Changes in immunophenotypic profiles of whole peripheral blood of animals infected with HTLV-1$_{WT}$ or HTLV-1$_{p12KO}$. (**A**) Schematic diagram of study design. Green arrows represent the day of treatment with M-T807R1, red arrows represent the day of Clodrosome administration, blue arrows indicate inoculation day of the lethally irradiated 729.6 cells lymphoblastoid B-cell lines producing HTLV-1$_{WT}$ or HTLV-1$_{p12KO}$ [24, 50]. The animal ID is given on the right of each timeline. Absolute counts of $CD4^+$, $CD8^+$, B-cells, and frequencies of $NKG2A^+$ cells, monocytes, $CD14^+CD16^-$, $CD14^+CD16^+$, $CD14^-CD16^+$, $CD4^+CD95^+$, $CD4^+CCR4^+$ cells and neutrophils before (baseline, B.), during (day 0), and after (weeks 1, 2, 3, 5, 7, 10, 12, 16, 21) the administration of M-T807R1 and Clodrosome in animals infected with (**B**) HTLV-1$_{WT}$ or (**C**) HTLV-1$_{p12KO}$. Asterisk (*) indicates $p < 0.05$ as calculated using student T-test.

Figure S3. Associations between the changes in frequency of cells and changes in cytokine/chemokine levels over time. Fold-changes in frequencies or counts of different cell populations: $CD8^+$ and $CD4^+$ T cells, B cells ($CD20^+$), monocytes and their subsets: classical ($CD14^+CD16^-$), intermediate ($CD14^+CD16^+$), and non-classical ($CD14^-CD16^+$), $NKG2a^+$ cells, neutrophils, $CD4^+CD95^-$, $CD4^+CD95^+$, and $CD4^+CCR4^+$ from whole blood were measured at baseline and throughout the study (weeks 2, 5, 7, 16 and 21), together with the levels of IFNγ, IL-8, IL-1β, IL-10, IL-6, IL-13, TNFα, IL-18, MIP-1β, sCD40L, and MIP-1α, measured in the plasma of HTLV-1$_{WT}$ or HTLV-1$_{p12KO}$ infected macaques. Correlation analyses were performed among these fold-changes using non-parametric Spearman rank correlation method separately for each virus group ($n = 5$, $n = 5$). White asterisks (*) indicate $p < 0.05$. Scale representing the Spearman's rank correlation coefficients (R) is given to the right of the plot. Positive correlation is shown in brown, negative correlation in purple. Larger size and brighter color of squares indicate larger R. (**A**) HTLV-1$_{WT}$ infected animals, (**B**) HTLV-1$_{p12KO}$ infected animals, (**C**) summary of the associations with $p < 0.05$.

Figure S4. HTLV-1$_{WT}$ and HTLV-1$_{p12KO}$ infectivity after re-depletion using M-T807R1/Clodrosome. (**A**) Schematic of study design. Green arrows represent the day of treatment with M-T807R1, red arrows represent the day of Clodrosome administration, and blue arrows indicate inoculation day of the lethally irradiated 729.6 cells lymphoblastoid B-cell lines producing HTLV-1$_{WT}$ or HTLV-1$_{p12KO}$. (**B**) The frequency of $CD8^+$ T-cells, monocytes, $NKG2a^+$ cells, and $CD4^+CCR4^+$ cells before (baseline, B. [week 28]), during (day 0), and after (weeks 29, 31, 33) the second administration of M-T807R1/Clodrosome in animals infected with HTLV-1$_{WT}$. (upper panels, red) or HTLV-1$_{p12KO}$ (lower panels, blue). Animal ID is given on the right of the graphs.

Figure S5. Viral DNA in PBMCs of HTLV-1$_{WT}$ and HTLV-1$_{p12KO}$ infected macaques after re-depletion. HTLV-1 proviral load (PVL) in PBMCs of infected animals belonging to (**A**) HTLV-1$_{WT}$ and (**B**) HTLV-1$_{p12KO}$ shown as percent. Heatmaps of nested PCR for the *gag* and *orf-I* genes in PBMCs of infected animals belonging to (**C**) HTLV-1$_{WT}$ and (**D**) HTLV-1$_{p12KO}$, collected after re-treatment with M-T807R1/Clodrosome at week 28 (baseline, B.), day 0, and weeks 29, 31, and 33. Genomic DNA was extracted to amplify viral DNA. The PCR products were sequenced to verify HTLV-1$_{p12KO}$ virus versus HTLV-1$_{WT}$. Positive amplification of either *gag* or *orf-I* by nested PCR is shown in red; absence of amplification is shown in yellow.

Figure S6. Associations between the changes in frequency of cells and changes in cytokine/chemokine levels after re-depletion with M-T807R1/Clodrosome. Fold-changes of the frequencies or counts of different cell populations: $CD8^+$, $CD4^+$ T cells, B cells ($CD20^+$), monocytes and their subsets: classical ($CD14^+CD16^-$), intermediate ($CD14^+CD16^+$) and non-classical ($CD14^-CD16^+$), $NKG2a^+$ cells, neutrophils, $CD4^+CD95^-$, $CD4^+CD95^+$, and $CD4^+CCR4^+$ from whole blood, were measured at the start of the study (baseline) and at various timepoints pre (week 28) and post-re-depletion (weeks 1, 3, and 5), together with the levels of IFNγ, IL-8, IL-1β, IL-10, IL-6, IL-13, TNFα, IL-18, MIP-1β, sCD40L, and MIP-1α, measured in the plasma of HTLV-1$_{WT}$ or HTLV-1$_{p12KO}$ infected macaques. Correlation analyses among these fold-changes were performed using non-parametric Spearman rank correlation method separately for each virus group ($n = 5$, $n = 5$). White asterisks (*) indicate $p < 0.05$. Scale representing the Spearman's rank correlation coefficients (R) is given to the right of the plot. Positive correlation is shown in brown, negative correlation in purple. Larger size and brighter color of squares indicate larger R. (**A**) HTLV-1$_{WT}$ infected animals, (**B**) HTLV-1$_{p12KO}$ infected animals, (**C**) summary of the associations with $p < 0.05$.

Figure S7. Viral dissemination in HTLV-1 infected animals treated with M-T807R1 or M-T807R1/Clodrosome prior to infection. (**A**) Heatmap of nested PCR of *orf-I* and (**B**) *gag* genes in DNA isolated from biopsies from the ileum, colon, thymus, skin, jejunum, cortex, lung, spleen, inguinal lymph nodes (LN), mesenteric LN, axillary LN, and lung LN, and BAL collected at the time of euthanasia following virus inoculation, together with PBMCs and bone marrow, from animals treated with M-T807R1 (CD8/NK) or M-T807R1/Clodrosome (CD8/NK/monocytes). Genomic DNA was extracted to amplify viral DNA. Nested PCR and qPCR were performed as described in Materials and Methods. The PCR products were sequenced to verify HTLV-1p12KO and HTLV-1WT sequences. Positive amplification of either *gag* or *orf-I* by nested PCR is shown in red; absence of amplification is represented in yellow. (**C**) The percent HTLV-1 proviral load (PVL), in tissues of infected animals belonging to HTLV-1WT and HTLV-1p12KO after the second treatment with M-T807R1/Clodrosome. HTLV-1 provirus DNA levels (PVL) were calculated by the following formula: (copies of HTLV-1 (pX)/(copies of RNase P/2)x100 cells.

Table S1. Animal prior history

Group	Treatment	Animal ID	Source	Treatment history	Viral exposure	Route of exposure	SIV/HSIV PCR
HTLV-1_{WT}	M-T807R1 antibody + Clodrosome®	15P044	*Covance Research Products* Inc. Alice, TX	A244-WT+ALVAC	SHIV-1157(QNE)Y173H	Intra-rectal	-
		18C266		A244-WT+ALVAC			-
		17P008		A244-DV1+ALVAC			-
		17P062		control			-
		17P039		control			-
HTLV-1_{p12KO}		17P002		A244-WT+ALVAC			-
		15P062		A244-DV1+ALVAC			-
		17P049		A244-DV1+ALVAC			-
		17P037		control			-
		17P030		control			-

Table S2. Antibodies used for non-human primate flow cytometry.

**Target**	**Clone**	**Fluorochrome**	**Source**
**Whole blood phenotyping**			
CD3	SP34-2	AlexaFluor 700	BD Biosciences
CD4	L200	BV750	BD Biosciences
CD8	SK1	BUV805	BD Biosciences
CD8	DK25	FITC	BD Biosciences
CD11b	D12	APC-Cy7	BD Biosciences
CD11c	B-ly6	BV711	BD Biosciences
CD14	M5E2	BB700	BD Biosciences
CD16	3G8	BV421	BD Biosciences
CD20	2H7	BV570	BioLegend
CD25	BC96	BUV737	ThermoFisher
CD28	CD28.2	BUV496	BD Biosciences
CD45	D058-1283	BV786	BD Biosciences
CD49d	9F10	BUV563	BD Biosciences
CD56	NCAM 16.2	BUV365	BD Biosciences
CD66abce	TET2	APC	Miltenyi Biotec
CD95	DX2	PE-Cy5	BioLegend
CD123	7G3	BV650	BD Biosciences
CD127	eBioRDR5	PE	ThermoFisher
CD159 (NKG2a)	Z199	PE-Cy7	Beckman Coulter
CD194 (CCR4)	1G1	BV605	BD Biosciences
HLA-DR	G-46-6	BUV661	BD Biosciences

Table S2. Antibodies used for non-human primate flow cytometry. Antibodies used for non-human primate flow cytometry, including clones, fluorochromes, and source.

Table S3. Cell frequency over time. Changes in phenotypic profiles of different cell populations in whole peripheral blood after first and second treatment with M-T807R1/Clodrosome of animals infected with HTLV-1_{WT} or HTLV-1_{p12KO}.

Table S4. Changes in cytokine/chemokine levels. Plasma from macaques were analyzed for changes in cytokine/chemokine levels over time after first and second treatment with M-T807R1/Clodrosome of animals infected with HTLV-1$_{WT}$ or HTLV-1$_{p12KO}$.
